# An intronic enhancer of *Bmp6* underlies evolved tooth gain in sticklebacks

**DOI:** 10.1371/journal.pgen.1007449

**Published:** 2018-06-14

**Authors:** Phillip A. Cleves, James C. Hart, Rachel M. Agoglia, Monica T. Jimenez, Priscilla A. Erickson, Linda Gai, Craig T. Miller

**Affiliations:** Department of Molecular and Cell Biology, University of California-Berkeley, Berkeley CA, United States of America; Fred Hutchinson Cancer Research Center, UNITED STATES

## Abstract

Threespine stickleback fish offer a powerful system to dissect the genetic basis of morphological evolution in nature. Marine sticklebacks have repeatedly invaded and adapted to numerous freshwater environments throughout the Northern hemisphere. In response to new diets in freshwater habitats, changes in craniofacial morphology, including heritable increases in tooth number, have evolved in derived freshwater populations. Using a combination of quantitative genetics and genome resequencing, here we fine-mapped a quantitative trait locus (QTL) regulating evolved tooth gain to a cluster of ten QTL-associated single nucleotide variants, all within intron four of *Bone Morphogenetic Protein 6* (*Bmp6*). Transgenic reporter assays revealed this intronic region contains a tooth enhancer. We induced mutations in *Bmp6*, revealing required roles for survival, growth, and tooth patterning. Transcriptional profiling of *Bmp6* mutant dental tissues identified significant downregulation of a set of genes whose orthologs were previously shown to be expressed in quiescent mouse hair stem cells. Collectively these data support a model where mutations within *a Bmp6* intronic tooth enhancer contribute to evolved tooth gain, and suggest that ancient shared genetic circuitry regulates the regeneration of diverse vertebrate epithelial appendages including mammalian hair and fish teeth.

## Author summary

Understanding how traits evolve in nature remains a fundamental goal in biology. Threespine stickleback fish offer a powerful system to address this question. Ancestral marine sticklebacks have colonized new freshwater environments, where new traits evolve, including increases in tooth number. This evolved increase in tooth number arises late in development and is associated with an accelerated tooth replacement rate in high-toothed freshwater fish. Using genetic and genomic data, here we mapped a genomic region regulating evolved tooth gain to an intronic region of *Bone Morphogenetic Protein 6* (*Bmp6*). This intronic region contains a transcriptional enhancer that drives gene expression during tooth development and replacement. We induced mutations in a coding region of *Bmp6* and found required roles for *Bmp6* for viability, growth, and tooth patterning. By comparing genome-wide gene expression data in wild-type and *Bmp6* mutant dental tissues, we found significant downregulation of a set of genes whose orthologs were previously shown to be expressed in quiescent mouse hair stem cells. Collectively these data support a model where mutations within *a Bmp6* intronic tooth enhancer contribute to evolved tooth gain, and suggest that different vertebrate epithelial appendages such as teeth and hair regenerate using an ancient shared genetic program.

## Introduction

Finding the genes and ultimately the mutations that drive the evolution of animal form remains an important goal in biology [1]. The *cis*-regulatory hypothesis proposes that *cis*-regulatory changes are the most frequent substrate for morphological evolution because these mutations are more likely to bypass the negative pleiotropy typically generated by coding mutations in developmental regulatory genes [2]. Although many studies in a variety of organisms have found *cis*-regulatory alleles underlying morphological evolution, less is known about why or how cis-regulatory alleles are used [3,4]. For example, for genes found to have *cis*-regulatory alleles associated with evolved differences, whether coding mutations generate negative pleiotropy and/or reduced fitness remains largely untested in many natural populations.

Teeth are a classic model system for studying organ development and evolution in vertebrates [5,6]. During tooth development, epithelial and mesenchymal cells reciprocally signal to each other, integrating dynamic BMP, TGF-β, FGF, SHH, Notch, Activin, EDA, and Wnt signals to orchestrate the formation of a mature tooth [7,8]. Bone Morphogenetic Protein (BMP) signaling plays multiple critical roles during tooth development. During tooth initiation, epithelial *Bmp4* inhibits expression of *Pax9* and *Pitx2*, developmental markers of the forming tooth placode [9,10]. These results suggest an inhibitory role of BMP signaling on tooth development. However, several lines of evidence support an activating role of BMPs on tooth development. For example, exogenous *Bmp4* can rescue tooth development in *Msx1* mutant mice and accelerate tooth development in cultured tooth mandibles, suggesting an activating role of BMP signaling [11,12]. Furthermore, mice with dental epithelial ablation of the BMP receptor, *Bmpr1a*, or transgenic for a construct overexpressing a BMP antagonist, *Noggin*, in dental epithelium have tooth arrest at the bud and placode stage, respectively [13,14]. Together, these results suggest that there are both activating and inhibitory roles of BMP signaling during tooth development. However, the roles of many BMP signaling components are not fully understood. Furthermore, the genetic pathways of early tooth pattern and initiation have been extensively studied and well characterized in mice. Because mice are monophyodont rodents that do not replace their teeth, considerably less is known about the developmental genetic basis of tooth replacement. Polyphyodont vertebrates (e.g. sharks, teleosts, and reptiles) that continuously replace their teeth offer an opportunity to study the genetic and developmental basis of tooth regeneration [6].

Threespine stickleback fish (*Gasterosteus aculeatus*) are an excellent model for understanding the molecular genetic basis of natural variation, including evolved differences in tooth number [15,16]. Sticklebacks have undergone a dramatic adaptive radiation in which ancestral marine sticklebacks have colonized freshwater lakes and streams throughout the Northern hemisphere [17]. Recent genetic studies have implicated *cis*-regulatory changes of developmental signaling molecules as underlying several aspects of stickleback morphological evolution [18–23]. Genome-wide searches for regions under selection during freshwater adaptation have found an enrichment in non-coding elements of the genome, further implicating *cis*-regulatory changes in underlying stickleback evolution [24].

Freshwater sticklebacks have evolved several morphological adaptations in their head skeleton, some likely due to the shift to feeding on larger prey in freshwater niches [25]. While many freshwater adaptations in sticklebacks involve skeletal loss, a constructive gain of pharyngeal tooth number is seen in freshwater benthic (adapted to lake bottom) and creek populations [19,26]. Pharyngeal teeth lie in the pharynx of fish and are serial and phylogenetic homologs of mammalian oral teeth [27]. Pharyngeal jaw patterning is an adaptive trait in fish that covaries with diet and ecological niche [28]. Many aspects of the developmental genetic circuitry regulating tooth development are conserved from mice to fish [29–31]. Thus, evolved tooth gain in sticklebacks provides a powerful opportunity to understand the evolutionary genetics of tooth development and replacement.

Evolved tooth gain in benthic freshwater fish from Paxton Lake in British Columbia is accompanied by an increase in the size of the tooth field, a decrease in tooth spacing, and an increase in tooth replacement rate late in development [19,26] (Table 1, columns 1–3). Previously we showed that this derived tooth pattern is partially explained by a large effect quantitative trait locus (QTL) on chromosome 21 that is associated with a late-acting *cis*-regulatory downregulation of *Bmp6* expression from benthic alleles in dental tissue [19] (Table 1, column 4). These results make *Bmp6* an excellent candidate gene for underlying evolved tooth gain by regulating tooth patterning and replacement. As no coding changes were found between marine and benthic freshwater alleles of *Bmp6* [19], we sought to map candidate regulatory regions of *Bmp6* associated with evolved tooth gain. Here, we use a combination of recombinant mapping, comparative genomics, genome editing, and transcriptional profiling to further dissect the molecular genetic basis of evolved tooth gain and the role of *Bmp6* during tooth development in threespine sticklebacks.

**Table 1:** Summary of phenotypes seen at different stages in wild, lab-reared, and *Bmp6* mutant benthic fish.

Stage	Tooth number in wild fish	Tooth number in lab-reared fish	*Bmp6* allele-specific expression in dental tissue	Tooth number in fish homozygous for induced *Bmp6* mutations	Tooth number in fish heterozygous for induced *Bmp6* mutations	Intron 4 enhancer spatiotemporal and/or quantitative differences
Larval/early juvenile	?	not diff.	not diff.	-	not diff.	?
Late juvenile	?	+	-	no data (lethal)	not diff.	?
Adult	+	+	-	no data (lethal)	-	?

Each row shows a developmental stage from a previous [19] or this study. Shown are the evolved benthic phenotypes relative to ancestral marine phenotypes (columns 2–4) and the mutant phenotype relative to wild-type phenotype (column 5–6). “+” = benthic phenotype was significantly higher than marine phenotype, “not diff.” = no significant differences were observed, “-” = benthic phenotype was significantly lower than marine phenotype (column 4) or mutant phenotype was significantly lower than wild-type phenotype (columns 5–6). “?” = unknown.

## Results

### Recombinant mapping of chromosome 21 tooth number QTL identifies an 884 kb interval containing *Bmp6*

**Fig 1:**
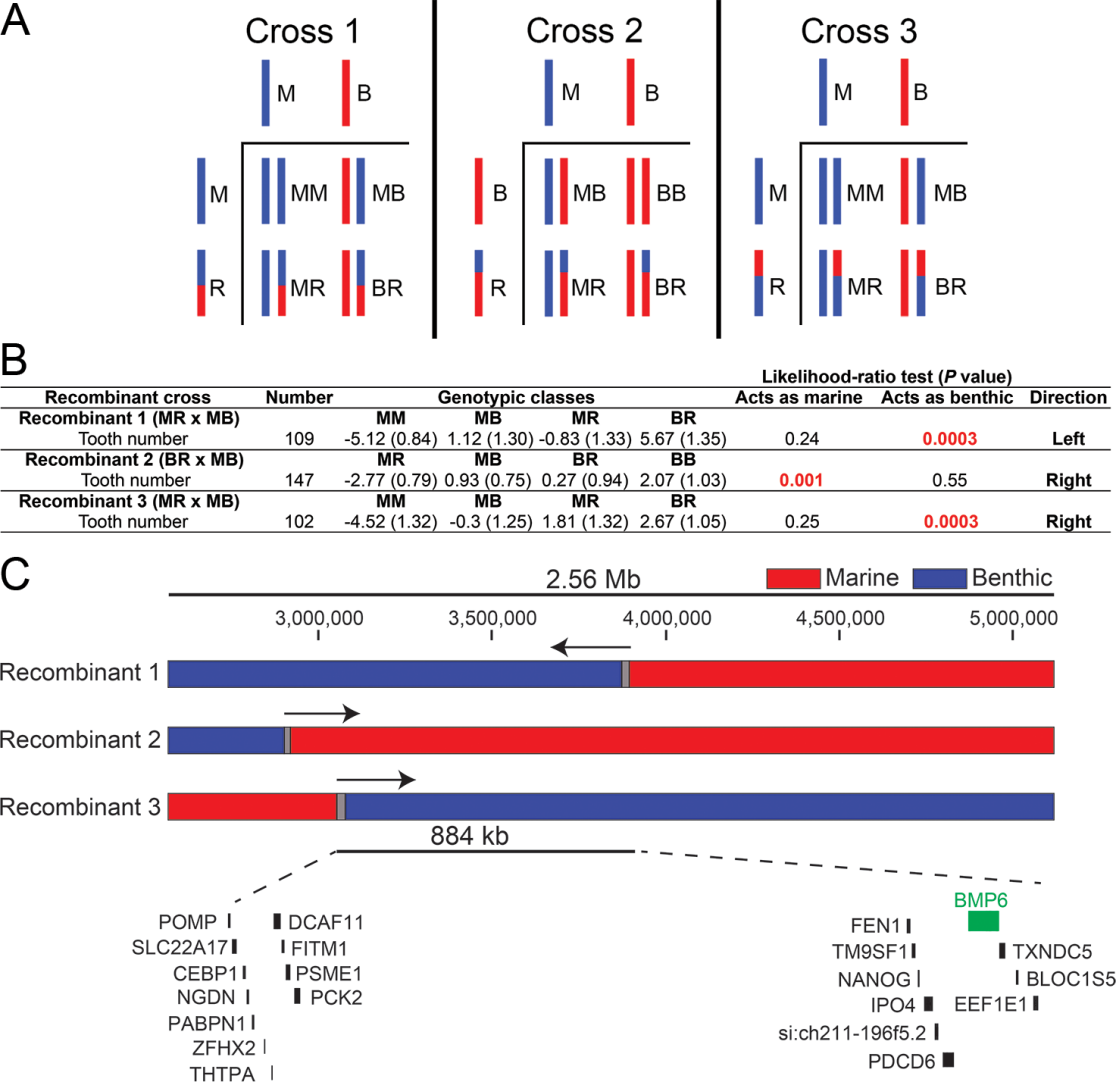
Recombinant mapping of chromosome 21 tooth QTL supports a 884 kb interval containing *Bmp6*. (A). Schematic of three recombinant crosses. In each cross, fish heterozygous for recombinant and marine (Cross 1 and 3) or recombinant and benthic (Cross 2) alleles of chromosome 21 were crossed to fish heterozygous for non-recombinant marine and benthic alleles of chromosome 21. For each cross, cartoons of Punnett squares are shown, with haploid genotypes to the left and top and four classes of resulting diploid genotypes shown in the lower right. (B) Size-corrected total ventral pharyngeal tooth number and standard error are listed for each genotypic class within each of the recombinant crosses. For each cross, parental genotypes of the tooth QTL are listed and coded: marine (M), benthic (B), or recombinant (R). Likelihood ratio tests were used to test whether recombinant chromosome effects on tooth number behaved like a marine or benthic chromosome (see Methods). *P*-values from each likelihood ratio test are listed with the supported direction column in B. (C) The chromosome 21 tooth QTL was previously fine mapped to a 2.56 Mb region containing *Bmp6* along with 58 other Ensembl predicted genes [19]. The three recombinant chromosome 21s tested are shown. Genotypes are colored red for marine, blue for benthic, and grey for unresolved. Arrows denote position of tooth QTL supported by each recombinant chromosome. The final recombinant mapped interval is 884 kb in the reference genome assembly and contains 21 predicted genes, including *Bmp6*.

We previously identified and fine-mapped a large effect tooth number QTL to a 2.56 Mb 1.5-LOD interval on stickleback chromosome 21 containing an excellent candidate gene, *Bone Morphogenetic Protein 6* (*Bmp6*), along with 58 other predicted genes [19,32]. To further fine-map this QTL, we identified three chromosomes with marine-benthic recombination events within the 2.56 Mb fine-mapped interval (Fig 1). Fish with each of these recombinant chromosomes were crossed to fish heterozygous for marine and benthic alleles of chromosome 21 to generate large (>100 fish each) crosses to test these recombinant chromosomes for effects on tooth number (Fig 1A, S1 Table). Recombinant chromosomes that increase tooth number compared to marine chromosomes suggest that the tooth controlling region of chromosome 21 lies within the benthic portion of the recombinant chromosome. We used a likelihood ratio test to determine whether each recombinant chromosome behaved more like a marine or benthic chromosome. Recombinant chromosomes one and three increased tooth number, each behaving like a benthic allele of chromosome 21 (*P* value from likelihood ratio test = 3.0 x 10^−4^ for both) (Fig 1B). Recombinant chromosome two did not increase tooth number, behaving like a marine allele of chromosome 21 (*P* = 1 x 10^−3^ from likelihood ratio test) (Fig 1B). Together, these recombinant crosses support a new smaller genetic interval, 884 kb in the stickleback reference genome assembly [24], that contains 21 predicted genes including *Bmp6* (Fig 1C), reducing the physical size of the interval and number of genes by 65% and 64%, respectively.

### Seven out of eight derived benthic chromosomes have a large effect tooth QTL

**Fig 2:**
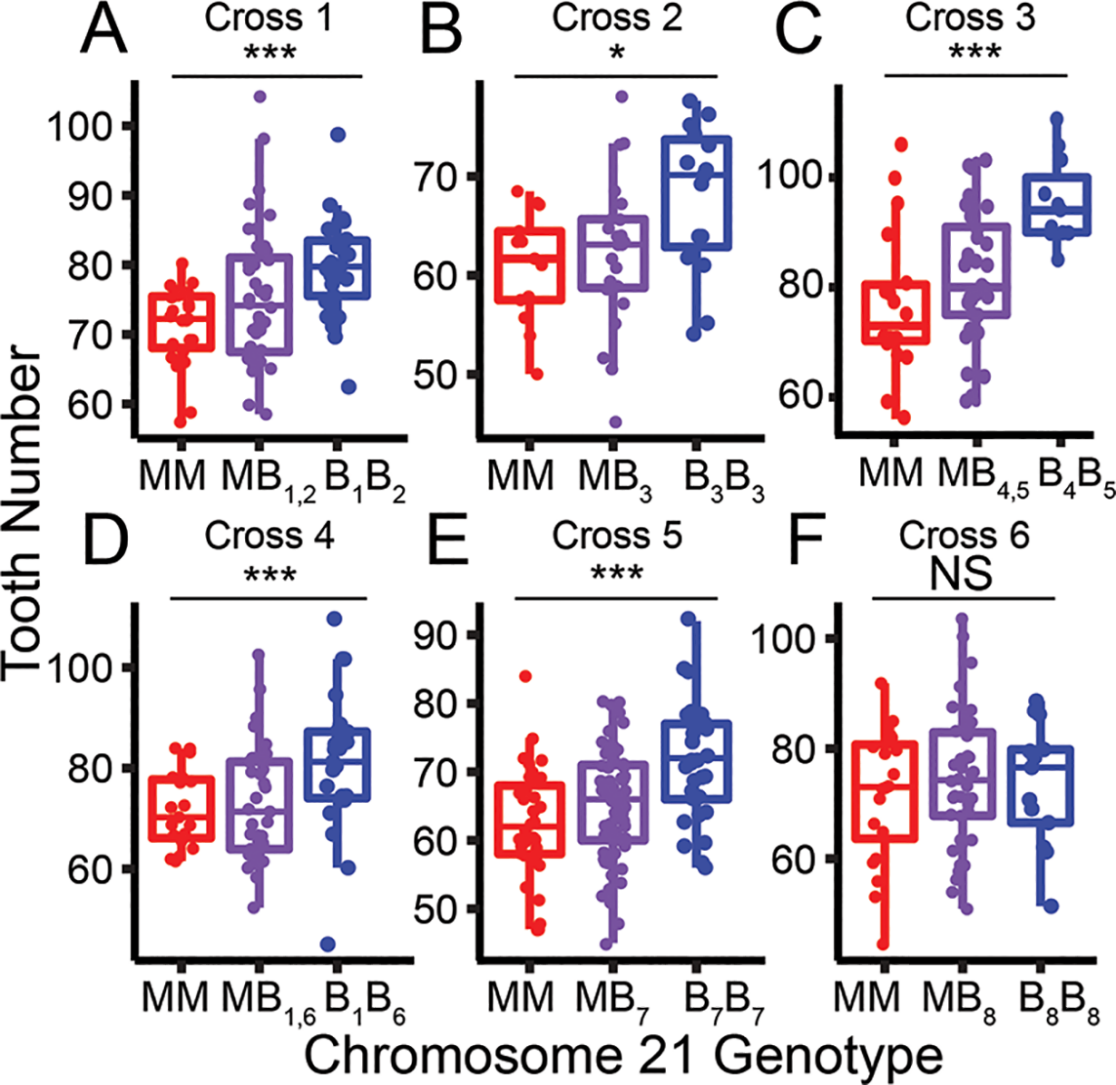
Seven of eight benthic chromosome 21s have a tooth QTL. (A-F) Results from six benthic by marine F2 crosses testing eight molecularly distinct (see Methods) benthic chromosome 21s (B_1-8_) are shown. (A-E) Benthic chromosomes 1–7 had strong effects on tooth number; however (F) the benthic chromosome 8 had no detectable effects on tooth number. Back-transformed total tooth numbers from marine homozygous (red), heterozygous (purple), and benthic homozygous (blue) fish for chromosome 21 are shown (see Methods). *P* values from an ANOVA for cross 1–6 are 0.002, 0.024, 0.0005, 0.004, 2.11x10^-5^, 0.69, respectively (* = *P <* 0.05, *** = *P* < 0.01). F2 crosses 1, 3, and 4 are testing two benthic chromosomes each and crosses 2, 5, and 6 each are testing one. Crosses 1 and 4 share a benthic chromosome. See S2 Table for more details.

To estimate the frequency of the chromosome 21 high tooth number allele within the wild Paxton benthic population, we generated six marine by benthic F2 crosses testing eight wild-derived benthic chromosomes (named B_1_-B_8_, Fig 2, S2 Table). These chromosomes had different genotypes at three microsatellite loci located 5’, within, and 3’ of the chromosome 21 tooth QTL, suggesting they are molecularly distinct wild chromosomes (S2 Table, see Methods). We found that seven of these eight benthic chromosomes had significant effects on tooth number with the same direction and similar magnitude of effect (Fig 2, S2 Table). The benthic chromosome tested in cross 6 (B_8_) had no effects on tooth number (Fig 2, S2 Table). These results together suggest that the high tooth number allele on chromosome 21 is at high frequency in the Paxton benthic population, but at least one lower-frequency benthic allele is not associated with an increase in tooth number.

### Whole genome resequencing reveals a cluster of QTL-associated variants in intron 4 of *Bmp6*

**Fig 3:**
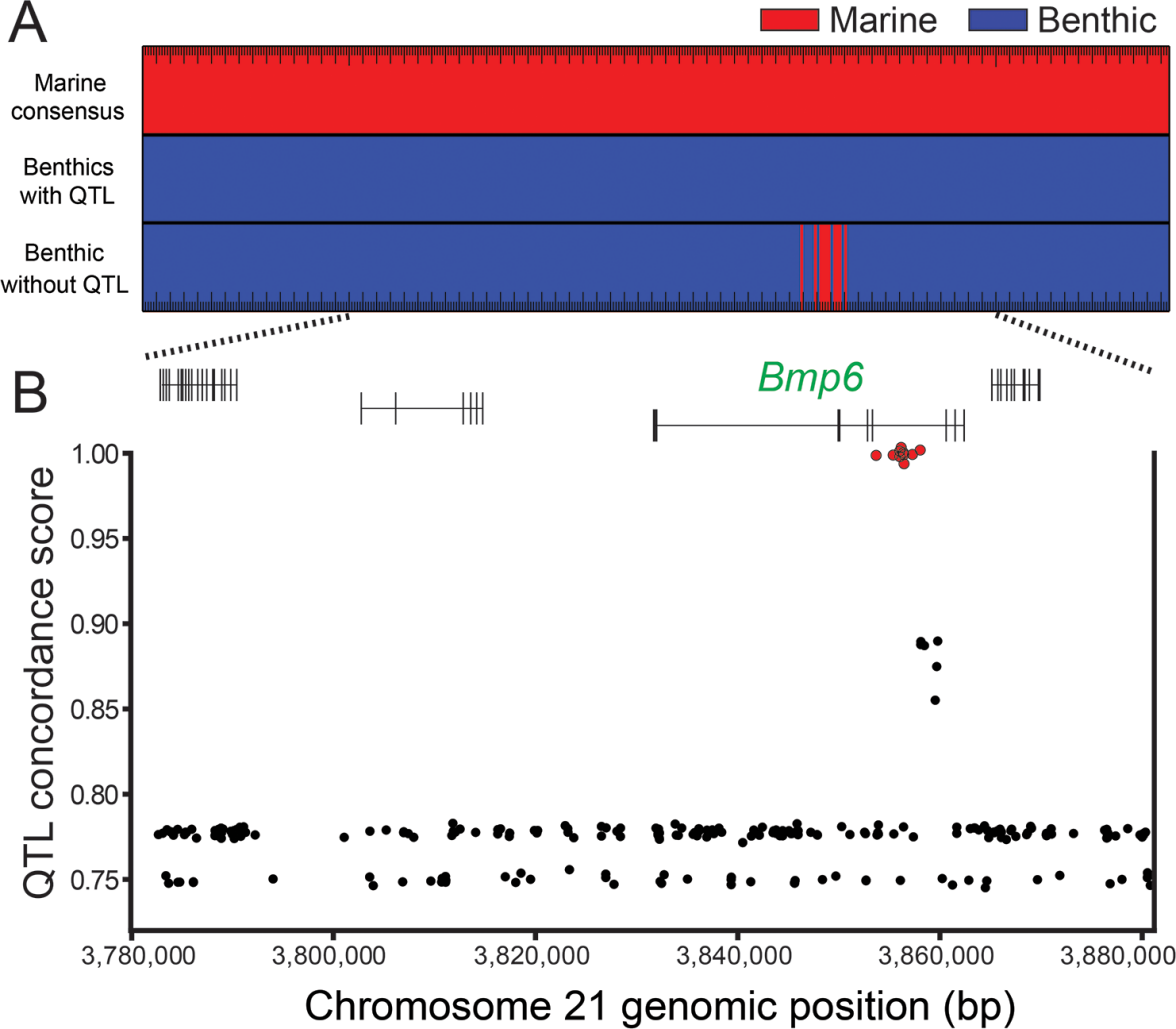
Comparative genomics reveal QTL-associated variants in intron 4 of *Bmp6*. (A) Comparing genomic sequences of the fine-mapped tooth QTL (from Fig 1C) between marine (n = 3, from crosses 2, 5, and 6) and benthic chromosomes with the tooth QTL (n = 7, from crosses 1–5) identified a set of variants with opposite homozygous genotypes, colored red for marine (top) and blue for benthic (middle). Note that only positions with opposite homozygous genotypes within this 884 kb are shown. The benthic chromosome without the QTL (chromosome B_8_ from cross 6) had a cluster of variants sharing the consensus marine genotype (bottom). (B) The ten variants with perfect QTL association (red points) all lie within intron 4 of *Bmp6*. The y-axis shows QTL concordance score (see Methods), a metric of concordance between genotype and presence or absence of tooth QTL. Gene model of *Bmp6* and surrounding genes are based on Ensembl predictions [24].

We hypothesized that the Paxton benthic chromosome 21 alleles that increase tooth number (B_1-7_, Fig 2) share sequence variants that underlie evolved tooth gain that are not present on marine alleles or the benthic chromosome 21 allele without the tooth QTL (B_8_, Fig 2). To test for QTL-associated variants, we resequenced the genomes of the four benthic grandparents from crosses 1–4, two F2 fish homozygous for chromosomes B_7_ and B_8_, and the three marine grandparents from crosses 2, 5, and 6 tested in Fig 2 (S3 Table). We identified 372 sequence variants (consisting of 323 SNPs, and 49 indels) within the 884 kb fine-mapped genetic interval that were present on all the benthic chromosomes with a large effect QTL, but not present on marine chromosomes (Fig 3A). We gave variants a QTL concordance score: the absolute value of the proportion of times a variant allele is found in the benthic fish with a chromosome 21 tooth QTL minus the proportion of times the same allele was found in fish without a tooth QTL. Only ten of these variants (all SNPs) were perfectly associated with the presence of the tooth QTL (Fig 3). Strikingly, all of these variants lie within a ~4.4 kb region of *Bmp6* intron 4 (Fig 3B, S4 Table).

### QTL-associated variants surround a tooth and fin enhancer in intron 4 of *Bmp6* that drives overlapping and distinct expression patterns as the *Bmp6* 5’ enhancer

We previously showed that a *cis*-regulatory decrease in expression of *Bmp6* is associated with the chromosome 21 tooth QTL in Paxton benthic fish, suggesting that changes to *Bmp6* regulatory elements underlie the tooth QTL [19]. We hypothesized that the region of intron 4 containing tooth QTL specific variants is a tooth enhancer of *Bmp6* (Fig 3B). To test for enhancer function, we cloned a ~2 kb intron 4 genomic fragment from marine fish into a reporter construct (S5 Table). Transgenic fish for this construct expressed GFP in the distal tips of developing pectoral and median fins at eight days post fertilization (dpf), and pharyngeal and oral teeth at 10 dpf (S1 Fig). These domains have been previously shown to be endogenous sites of *Bmp6* expression in developing sticklebacks [19,33]. These results demonstrate that the fourth intron of *Bmp6* contains an enhancer active in developing teeth and fins.

**Fig 4:**
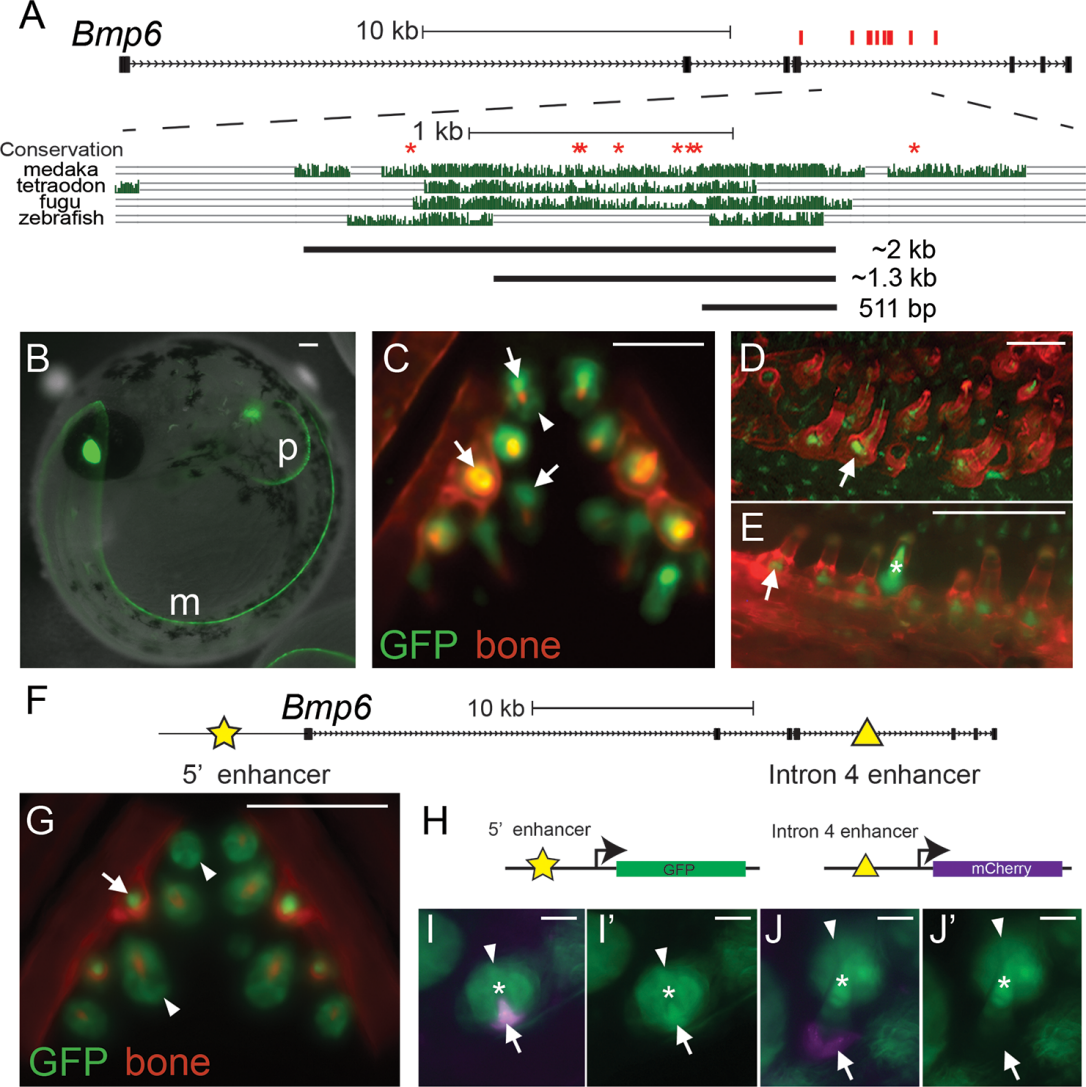
Intron 4 region with QTL-associated variants contains a tooth and fin enhancer. (A) Schematic of *Bmp6* locus. All ten QTL-associated variants (red ticks) are located within intron 4. Eight of these variants (red asterisks) are in conserved sequence, expanded below. Conservation in teleosts is shown from the UCSC genome browser (http://genome.ucsc.edu/). Black bars show the ~2 kb, 1.3 kb, and the 511 bp enhancer subclones tested. (B-E) GFP reporter expression from the 511 bp enhancer in stable transgenic fish. (B) At eight days post fertilization (dpf), expression was detected in the developing distal edge of the pectoral (“p”) and median fin (“m”). (C) By 13 dpf, relatively faint GFP expression was present in developing tooth epithelia (arrowhead) and stronger GFP expression was present in mesenchyme (arrows) of early stage and fully-formed teeth (see Figure S4 in [19] for time course of tooth epithelia and mesenchyme morphology in whole mounts). By late juvenile stages, mesenchymal expression was detected in developing pharyngeal (D) and oral (E) teeth. (F) The 5’ (star) and intron 4 (triangle) tooth enhancers of *Bmp6* are shown. (G) The previously described 190 bp 5’ *Bmp6* tooth enhancer [33] drove overlapping but distinct expression than the intronic enhancer. Compared to the intronic enhancer (C), the 5’ enhancer drove more persistent expression in tooth epithelial cells (arrowheads), and expression in tooth mesenchyme (arrow). (H) Fish doubly transgenic for the 190 bp 5’ enhancer driving GFP (green) and the 511 bp intron 4 enhancer driving mCherry (magenta) allow enhancer patterns to be directly compared. (I-I’) At early stages of tooth development, both enhancers drove mesenchymal expression (arrows), while the 5’ enhancer, but not the intron 4 enhancer, drove strong epithelial expression (arrowheads). (J, J’) As tooth development progresses, the intron 4 mesenchymal expression (arrow) extended to the base of the developing tooth, in cells not expressing the 5’ enhancer, while the 5’ enhancer continued to drive expression in both epithelial and mesenchymal cells. I and J show both GFP and mCherry channels overlaid, I’ and J’ show the GFP channels only. White asterisks in I, I’, J, and J’ mark mineralized teeth. Bone is counterstained with Alizarin Red in (C-E, G). Scale bars are 100 μm (B-G) and 10 μm (I-J).

To define the minimally sufficient enhancer, we subcloned the ~2 kb fragment into two smaller fragments of ~1.3 kb and 511 bp based on patterns of sequence conservation (Fig 4A, S5 Table), and tested for enhancer function in marine stickleback fish. The 511 bp construct is highly conserved in fish and contains no QTL-specific variants. The 1.3 kb construct includes the 511 bp region and a less conserved region that contains 6 of the 10 QTL-specific variants. The ~800 bp included in the ~1.3 kb construct but not the 511 bp construct drove no consistent expression, and no convincing differences were observed either between the ~1.3 kb construct and the 511 bp construct, or marine and benthic versions of the ~1.3 kb construct at early embryonic and larval stages [n > 3 injection rounds each, n > 20 GFP+ lenses (the internal control domain driven by the *Hsp70l* promoter) for both early embryonic and early larval comparisons]. Both the larger 1.3 kb construct and the 511 bp construct drove expression in the distal edges of the median and pectoral fins at eight dpf (Fig 4B). By 13 dpf, the 511 bp enhancer drove expression in mesenchymal cells in developing pharyngeal teeth, as well as expression in the tooth epithelium (Fig 4C). In developing teeth, the GFP-positive mesenchymal domain extended from each tooth germ deep into the tooth plate (Fig 4C). This tooth expression continued into late juvenile stages when the pharyngeal tooth number differences arise between marine and freshwater populations (Fig 4D) [19]. GFP expression was also detected in late juvenile oral teeth (Fig 4E). These results demonstrate that the intron 4 tooth QTL-associated variants surround an enhancer sufficient to drive expression in developing fins and teeth.

We previously identified a TGFβ-responsive 5’ enhancer of *Bmp6* that also drives expression in developing teeth and fins in sticklebacks [33]. Because stickleback *Bmp6* expression is spatially and temporally complex in developing teeth [19], we hypothesized that the two regulatory elements may control distinct aspects of *Bmp6* expression in teeth. To test this hypothesis, we compared GFP expression patterns in fish stably transgenic for reporter genes for the 190 bp 5’ tooth enhancer or the 511 bp intron 4 tooth enhancer (Fig 4C, 4F and 4G). As previously described [33], we found that the 5’ enhancer drives robust expression in developing tooth epithelium and adjacent tooth mesenchyme (Fig 4G). We found that the intron 4 enhancer drove expression that appeared distinct from the 5’ enhancer at some stages of tooth development (Fig 4C and 4G). The intronic enhancer drove expression in the mesenchymal cores of mature teeth similar to the expression driven by the 5’ enhancer. However, the intronic enhancer drove deeper mesenchymal expression around the base of the developing tooth compared to the 5’ enhancer (Fig 4C and 4G).

To directly compare the tooth expression domains driven by the two enhancers, we generated fish transgenic for both a 511 bp intron 4 enhancer mCherry reporter construct as well as a 190 bp 5’ tooth enhancer [33] GFP reporter construct (Fig 4H). The tooth expression domains were partially overlapping between the two enhancers in developing teeth (Fig 4I and 4J). As was seen comparing the stable lines, both enhancers drive similar mesenchymal expression at early stages of tooth development, but the 5’ enhancer and not the intron 4 enhancer drove strong epithelial expression at these stages (Fig 4I and 4I’). As tooth development progresses, the intron 4 enhancer drove expression at the base of the mineralized tooth, in mesenchymal cells that did not express the 5’ enhancer (Fig 4J and 4J’). These results suggest that *Bmp6* expression in tooth epithelial and mesenchymal cells is driven by at least two enhancers that drive partially overlapping yet distinct expression patterns.

### Induced mutations in stickleback *Bmp6*

**Fig 5:**
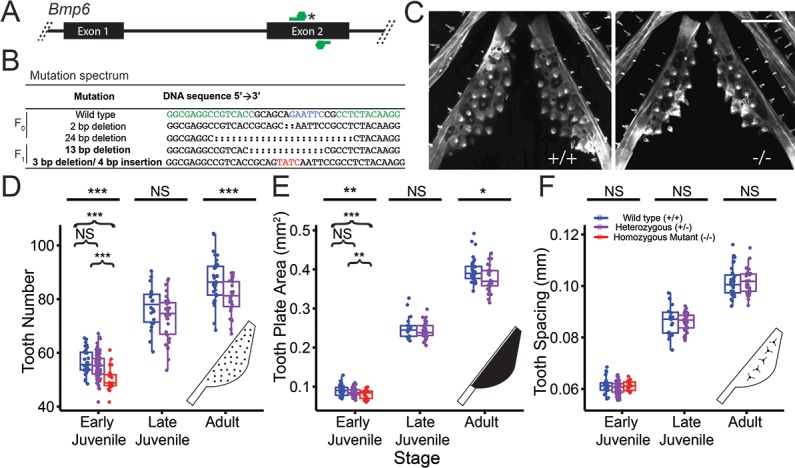
*Bmp6* is required for viability, growth, and tooth patterning. (A) Schematic of TALEN pair (green) targeting an *EcoR*I site (asterisk) in the second exon of *Bmp6*. (B) Sanger sequencing of F_0_ or F_1_ fish revealed a spectrum of genomic deletions (colons) and insertions (red) in *Bmp6*. The two mutations used in this study are in bold. In the wild-type sequence, the *EcoR*I site is shown in blue and the edges of the TALEN targeting sequences shown in green. (C) Confocal images of early juvenile (16–17 mm total length) wild-type (left) and homozygous mutant (right) ventral pharyngeal tooth plates showing fewer teeth in mutant. Mutant shown is transheterozygote for 13 bp deletion and 3 bp deletion+4 bp insertion. Scale bar is 200 μm. (D-F) Developmental time course of tooth number (D), tooth plate area (E), and tooth spacing (F) in wild-type (blue), heterozygous (purple), and homozygous mutant (red) fish. (D-E) Homozygous fish have recessive reduction of tooth number and tooth plate area at the early juvenile stage (Tukey post-hoc *P* values comparing wild-type to homozygous mutant are 9.3 x10^-6^ and 0.004, respectively and comparing heterozygous to homozygous mutant are 1.3x10^-4^ and 0.08, respectively). Tooth number and area diverges late in development between wild-type and heterozygous fish. (F) Tooth spacing is not significantly different in the mutant at any stage. The late juvenile and adult crosses were heterozygous mutant backcrossed to wild-type fish. For D-F, homozygous mutants include both fish homozygous for the 13 bp deletion mutation and fish transheterozygous for the 13 bp deletion and the 3 bp deletion + 4 bp insertion (see S7 Table and S1 File Source Data File).

To test whether *Bmp6* is required for tooth patterning in sticklebacks, we used transcription activator-like effector nucleases (TALENs) to generate two predicted loss-of-function mutations in stickleback *Bmp6* (Fig 5A, S6 Table). We designed a TALEN pair to target the highly conserved second exon of *Bmp6*, which is 5’ to the exons encoding the predicted secreted ligand. Thus early stop codons would be predicted to generate strong loss-of-function alleles. Injection of these TALEN RNAs into stickleback embryos efficiently induced mutations in the *Bmp6* target sequence. To identify mutations in F_0_ injected fish and mutations transmitted through the germline in F_1_ fish, we PCR amplified the surrounding sequence around the target site, digested this amplicon with *EcoR*I, then gel extracted and sequenced the uncut band (S2 Fig). We found that 24–57% of injected F_0_ stickleback embryos had detectable deletions, with up to 12% of these embryos appearing to have biallelic mutations (S2 Fig). Consistent with previous studies using TALENs in fish, we identified a spectrum of insertions and deletions at the target site (Fig 5B) [34]. We generated two mutant alleles that we bred to test for phenotypes: (1) a 13 bp deletion, and (2) a 3 bp deletion plus 4 bp insertion (Fig 5B bold). Both of these mutations are predicted to produce frameshifts and an early stop codon 5’ to the secreted BMP ligand and thus are both likely strong loss-of-function alleles (S3 Fig).

### *Bmp6* is required for viability, growth and tooth patterning

To test for tooth patterning phenotypes in *Bmp6* mutants, we intercrossed fish that were heterozygous for the 13 bp deletion or the 3 bp deletion plus 4 bp insertion and raised developmental time courses. Homozygous mutants were underrepresented from expected ratios at later developmental stages, suggesting early juvenile lethality (S7 Table). The surviving homozygous mutants tended to be slightly smaller (S7 Table). Because of the late stage lethality, we continued the *Bmp6* mutant time course with heterozygous backcrosses for late juvenile and adult stages. To test for required roles of *Bmp6* in tooth patterning, we quantified ventral pharyngeal tooth number, tooth plate area (size of tooth field), and inter-tooth spacing, three phenotypes controlled by the chromosome 21 tooth QTL [19] in the *Bmp6* mutant time course (Fig 5C–5F). At the early juvenile stage, homozygous mutants had a reduction of both tooth number and tooth plate area compared to wild-type or heterozygous fish (Fig 5D and 5E; Table 1, column 5). Beginning in early juveniles, heterozygous fish had fewer ventral teeth and smaller tooth plate area, which were both significantly more reduced at later time points including adults (Fig 5D and 5E; Table 1, column 6). There were no significant differences in inter-tooth spacing at any stage (Fig 5F). These results show that *Bmp6* is required for specifying tooth number and the size of the tooth field. To test whether fish transheterozygous for both the 13 bp deletion and the 3 bp deletion/4 bp insertion have tooth patterning phenotypes, we generated a transheterozygote cross using the two different *Bmp6* mutant alleles. We found that fish transheterozygous for the two different mutations had similar tooth patterning phenotypes as fish homozygous for the 13 bp deletion (S8 Table).

In addition to the bilateral ventral tooth plates, stickleback pharyngeal teeth are also present on two bilateral dorsal tooth plates, dorsal tooth plate 1 (DTP1) and dorsal tooth plate 2 (DTP2) [35]. We next asked whether *Bmp6* also regulates dorsal pharyngeal tooth number. We found no significant differences in tooth number of either dorsal tooth plate at early developmental stages (S4 Fig). In adults, DTP2 tooth number was significantly lower in heterozygous mutants, but to a lesser degree than the ventral tooth number differences at the same stage (S4 Fig). For both dorsal tooth plates, tooth numbers trended in the same direction as seen for the ventral tooth plates, with fewer teeth in mutants than wild types. These results demonstrate that, like the chromosome 21 tooth QTL [32], *Bmp6* dosage has stronger effects on ventral pharyngeal tooth number than dorsal pharyngeal tooth number.

### *Bmp6* regulates orthologs of BMP target genes, genes in the TGF-β signaling pathway, and genes upregulated in mouse hair follicle stem cells

**Fig 6:**
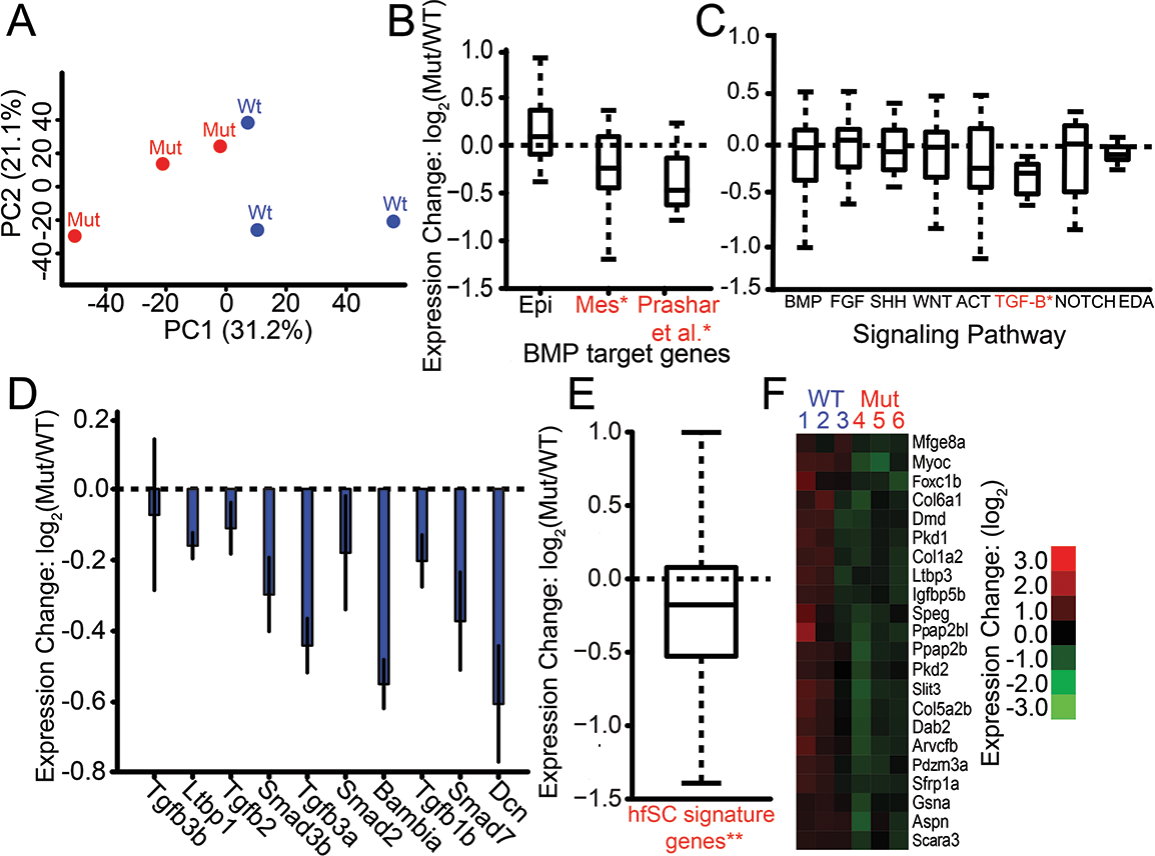
Transcriptional profiling reveals TGF-β signaling components, BMP target genes, and hair follicle stem cell signature genes are downregulated in *Bmp6* mutant tooth plates. (A) Principal component analysis of genome-wide expression levels in late juvenile ventral pharyngeal tooth plate tissue by RNA-seq separates wild-type (Wt, blue) and *Bmp6* mutants (Mut, red) along PC1. (B) BMP target genes in developing tooth epithelium and mesenchyme [8] were not affected (left bar), and significantly downregulated (*P* = 1.25 x 10^−2^, middle bar), in the mutant, respectively. A set of BMP target genes [36] was significantly downregulated in mutants (*P* = 3.12 x 10^−4^, right bar). (C) Expression of ToothCODE signaling pathways. Homozygous mutant fish (Mut) had significantly lower TGF-β pathway expression compared to wild-type fish (WT) (*P* = 4.7 x 10^−3^). None of the other pathways showed significant differences. (D) Each of the ToothCODE TGF-β genes was downregulated in the mutant. Error bars are SE of the mean. (E) A previously described set of genes upregulated in the mouse hair follicle stem cell niche [46] was downregulated in *Bmp6* mutants (*P* = 8.5 x 10^−12^). hfSC = hair follicle stem cells (F) Hair follicle stem cell signature genes showing significant downregulated expression in *Bmp6* mutants. See S1 File for gene expression levels and gene sets. For B, C, and E, gene sets with significant expression differences between wild-type and mutant are listed in red and with an asterisk.

To begin to identify the genetic networks downstream of *Bmp6*, we performed RNA-seq of early juvenile wild-type and 13 bp deletion homozygous mutant bilateral pharyngeal tooth plates (n = 3 of each genotype, S9 Table). Following read mapping and gene expression quantification, we performed principal component analysis of normalized read count of the entire dataset (Fig 6A). PC1 explains a large fraction of the total variance (31.15%), and discriminates between the *Bmp6* homozygous wild-type and mutant samples (Fig 6A). Furthermore, genes whose expression correlated with the first principal component were highly enriched for gene ontology terms related to development and cell signaling (S10 Table).

To test whether stickleback *Bmp6* regulates BMP target genes found in other systems, we compared the genes that were differentially expressed between wild-types and mutants to three different data sets, two from ToothCODE [8] and the third from a microarray study [36]. By combining literature mining of published mouse tooth development studies as well as their own functional analyses, the ToothCODE project collected a list of target genes downstream of BMP signaling in developing tooth epithelium or mesenchyme [8]. We tested whether stickleback orthologs of these epithelial and mesenchymal BMP target gene sets were differentially affected in *Bmp6* mutant tooth plate tissue. Orthologs of mesenchymal BMP target genes as a whole displayed significantly reduced expression in *Bmp6* mutants (*P* = 1.25 x 10^−2^), while orthologs of epithelial BMP target genes were not significantly affected (Fig 6B). A third set of BMP signaling target genes was identified in a meta-analysis of published microarray studies [36]. We next asked whether stickleback orthologs of this gene set were significantly downregulated in *Bmp6* mutant tooth plate tissue. We found this set of orthologs was significantly downregulated in *Bmp6* mutants (*P* = 3.12 x 10^−4^), with 15/17 displaying a lower mean expression (Fig 6B). These results show that stickleback *Bmp6* is required to regulate a conserved battery of BMP-responsive genes.

We hypothesized that the *Bmp6* tooth number phenotype may result from changes in major signaling pathways known to be involved in tooth development [6,7]. The ToothCODE project manually curated a list of genes involved in tooth development in eight major signaling pathways (BMP, FGF, SHH, Wnt, Activin, TGF-β, Notch, and EDA) important for tooth development in mice [8]. We asked whether stickleback genes annotated as being in each of these pathways were concertedly differentially expressed in *Bmp6* mutants compared to wild types. We found the TGF-β signaling pathway to be significantly downregulated (*P* = 4.7 x 10^−3^) in *Bmp6* mutant tooth plates (Fig 6C). Strikingly, all eight TGF-β components tested had reduced mean expression in *Bmp6* mutant tooth plates (Fig 6D). In contrast, none of the other seven signaling pathways had significant expression differences (Fig 6C), despite the differences in tooth number in *Bmp6* mutants. Together these data suggest that *Bmp6* positively regulates TGF-β signaling in stickleback tooth plate tissue.

In polyphyodont sharks, fish, reptiles, and mammals, *Sox2* has been implicated in putative epithelial stem cells during tooth replacement [37–39]. We found no significant differences in *Sox2* expression between *Bmp6* wild-type and mutant fish [mean FPKMs (Fragments Per Kilobase of transcript per Million mapped reads) of 91 and 97, respectively]. In mice, *Bmp6* inhibits the proliferation of hair follicle stem cells [40,41]. Teeth and hair are epithelial appendages with deep developmental and genetic homology [42–45]. Thus, we hypothesized that *Bmp6* may play a conserved role of mediating stem cell quiescence during tooth replacement. A previous study characterized a set of hair follicle stem cell signature genes that are upregulated in the stem cell niche in the mouse hair follicle relative to the proliferating hair germ [46]. *Bmp6* mutants showed a highly significant (*P* = 8.5 x 10^−12^) decrease in the expression of stickleback orthologs of these genes (Fig 6E and 6F). The reduced expression of the orthologs of these hair follicle stem cell signature genes supports the hypothesis that *Bmp6* regulates stem cell quiescence during tooth replacement.

## Discussion

### Mapping an evolved tooth gain QTL to a *Bmp6* intronic enhancer

We previously identified a *cis*-regulatory downregulation of *Bmp6* associated with the chromosome 21 tooth QTL [19]. Because there are no reported coding changes between marine and freshwater benthic alleles of *Bmp6* in wild sticklebacks [19], regulatory changes that change the spatiotemporal pattern and/or the quantitative levels of *Bmp6* expression likely modulate natural variation in tooth patterning. Here we combined recombinant mapping and comparative genomics of multiple QTL crosses to fine-map this chromosome 21 tooth QTL to a haplotype within the fourth intron of *Bmp6*. The association of ten variants in intron 4 of *Bmp6* with the chromosome 21 tooth QTL, together with our data showing intron 4 contains a robust tooth enhancer, suggest a model in which these QTL-associated variants at least partially underlie the tooth QTL. Although all of the tooth QTL-associated mutations are outside of the minimally sufficient 511 bp tooth enhancer, we propose that some or all of these variants underlie the *cis*-regulatory changes in *Bmp6*. One of the most outstanding questions for future research to address is whether these ten variants affect the spatiotemporal patterns and/or quantitative levels of enhancer activity (Table 1, column 7). Although comparing the ~1.3 kb marine and benthic constructs has revealed no obvious differences at early embryonic and larval stages to date, several technical challenges including mosaicism in F_0_s and position effects in stable lines make comparing two enhancers in different fish difficult. Due to the dynamic and complex expression patterns of the intronic enhancer, addressing potential marine/benthic enhancer differences would be facilitated by better tools to precisely compare enhancer activity, either at the same integration site using transgene landing pads, or in the same fish using bicistronic constructs separated by an insulator.

We note that the minimally sufficient 511 tooth enhancer contains a predicted Foxc1 binding site [47]. In mice, *Foxc1* regulates mammalian hair regeneration in part through regulating BMP signaling and appears to directly regulate *Bmp6* [48], so potential FoxC inputs into *Bmp6* expression in replacement teeth are especially intriguing. Of the marine/freshwater differences in the enhancer, one SNP alters a predicted NFATc1 binding site, a critical regulator of stem cell quiescence in the mouse hair follicle stem cell niche [49]. Another SNP affects a predicted Gli binding site, of interest because *Gli* expression is seen in multiple epithelial appendage stem cell niches in mice [50]. Future experiments will dissect what signals regulate this intronic enhancer, as well as what phenotypic consequences, if any, result from mutations in this enhancer.

This intronic enhancer, like the 5’ tooth enhancer [33], also drives embryonic and larval expression in developing pectoral and median fins. One interesting hypothesis these expression domains in fins raise is whether evolved differences in median or pectoral fin morphology are also regulated by this derived intronic haplotype. Perhaps supporting this hypothesis, a QTL regulating median fin morphology (dorsal spine 3 length) was previously mapped to a broad region of chromosome 21 overlapping *Bmp6* [32]. Future experiments will also test whether the marine and freshwater versions of the enhancer have different activity in fins.

### Regulation of *Bmp6* during tooth development and replacement

Our transgenic assays show that the intronic enhancer of *Bmp6* drives both overlapping and distinct domains of expression as the previously characterized 5’ *Bmp6* enhancer [33]. Both enhancers drive overlapping expression in the mesenchymal cores of developing teeth. However, relative to the 5’ enhancer, the intronic enhancer also appears to drive deeper and broader mesenchymal expression and more restricted epithelial expression. These differences in expression patterns from the two enhancers suggest different signaling inputs control the mesenchymal and epithelial expression of *Bmp6* in developing teeth. Our finding that the 5’ and intronic *Bmp6* enhancers drive partially non-overlapping expression patterns is reminiscent of the mouse *Bmp5* gene, which has two rib enhancers that drive expression in largely complementary patterns [51]. A modular *cis*-regulatory architecture is likely a common feature of *Bmp* genes, and could predispose these genes to frequently be used in morphological evolution [21,52–54].

### Required roles for *Bmp6* in survival, growth, and tooth patterning

This QTL confers late-acting (juvenile stage, >25mm in fish length) increases in tooth number and tooth field size, and decreases in tooth spacing [19]. Here we generated fish with induced mutations in *Bmp6* to directly test whether *Bmp6* played any required role in regulating tooth patterning. Strikingly, fish heterozygous for induced mutations in *Bmp6* also had developmentally late differences in tooth number and tooth field size, similar to the tooth QTL (Table 1). A second phenotypic similarity between the tooth QTL and induced mutations in *Bmp6* is a stronger effect on ventral tooth number than dorsal tooth number [19,32]. However, the direction of the *cis*-regulatory allele, where the high-toothed allele drives reduced *Bmp6* expression in *cis* relative to a marine allele [19], would predict that a mutation that lowers *Bmp6* mRNA levels would increase tooth number, while the *Bmp6* coding mutants have fewer teeth (Table 1). One explanation for this unexpected direction of effect could be a threshold effect: the *Bmp6* mutations were made in a freshwater benthic genetic background with already reduced levels of *Bmp6* expression, and further lowering of *Bmp6* activity could inhibit tooth development. One test of this model could be to analyze the role of *Bmp6* during tooth development in marine sticklebacks, or in other freshwater populations lacking the benthic *Bmp6* intronic haplotype reported here. Alternatively, the induced mutant coding alleles of *Bmp6* might not recapitulate the evolved *cis*-regulatory differences between marine and freshwater fish. The dynamic expression of *Bmp6* in dental epithelium and mesenchyme at different stages of tooth development is controlled by at least two different *cis*-regulatory elements ([33]; this study), which we show here drive expression at some stages in non-overlapping patterns. The evolved *cis*-regulatory allele of *Bmp6* may change the spatiotemporal pattern and/or levels of *Bmp6* mRNA in different tissues, leading to different phenotypes than the coding mutations. Inducing loss-of-function mutations in the two known stickleback *Bmp6* enhancers and assessing potential changes in tooth patterning could test this hypothesis.

The *cis*-regulatory hypothesis proposes that morphological evolution typically proceeds through *cis*-regulatory mutations that avoid the negative pleiotropy typical of coding mutations [1,2,55]. Recent studies have shown that *cis*-regulatory and coding mutations can drive morphological evolution, and that the type of mutation may depend on the degree of pleiotropy of the gene of interest [18,19,56,57]. The lethality and smaller size of fish homozygous for *Bmp6* coding mutations could explain why *cis*-regulatory changes of *Bmp6* have been used to evolve increases in tooth number.

There were no significant differences in tooth pattern at early developmental stages between wild-type and heterozygous *Bmp6* mutant fish. However, as these heterozygous fish continued to develop to adult stages, when newly forming teeth are likely replacement teeth, the reduction of tooth number and tooth plate area became more dramatic, suggesting that tooth development at late stages is more sensitive to the dosage of *Bmp6*. These differences could be due to different developmental or genetic constraints at the early juvenile and late adult stages of tooth patterning. For example, there could be more functional redundancy of *Bmp6* with other BMP ligands in teeth at early developmental stages that compensate in *Bmp6* heterozygous mutants. Alternatively, these differences may signify differing roles of *Bmp6* in primary and replacement tooth formation: later developing replacement teeth may be more sensitive to *Bmp6* dosage than primary teeth. However, homozygous mutants had significantly fewer teeth at early juvenile stages, suggesting *Bmp6* is also required for formation of primary teeth.

### Downstream targets of *Bmp6* signaling in dental tissue

To test which genes and pathways are downstream of *Bmp6* signaling, we used RNA-seq to compare genome-wide transcriptional profiles of wild-type and homozygous mutant *Bmp6* tooth plates. Seven signaling pathways were not significantly different in this contrast, perhaps surprising given the predicted difference in total tooth number in these samples. However, we found that there is a concerted downregulation of the TGF-β signaling pathway components in homozygous mutants. TGF-β signaling is required for tooth development [58–60]. Furthermore, TGF-β signaling regulates *Bmp6* expression in stickleback teeth through the previously described 5’ tooth enhancer [33]. These results suggest that TGF-β signaling is involved both upstream and downstream of *Bmp6* during tooth development.

During tooth development in mice, reciprocal signaling events involving *Bmp4* and *Msx1* occur between developing tooth epithelium and mesenchyme: *Bmp4* expression is first detected in dental epithelium, is required to induce *Msx1* expression in underlying mesenchyme, which in turn is required to induce *Bmp4* expression in dental mesenchyme [11,61–63]. Thus, *Bmp4* is thought to play critical roles during tooth development in both dental epithelium and mesenchyme. A large mouse gene expression study revealed sets of genes regulated by *Bmp2/4/7* in dental epithelium and mesenchyme [8]. We hypothesized that mouse BMPs and stickleback *Bmp6* regulate a conserved set of downstream genes in developing teeth. We tested this hypothesis by asking whether orthologs of known mouse BMP signaling target genes are differentially regulated in stickleback *Bmp6* mutant tooth plate tissue. Surprisingly, we found significantly reduced expression of the set of genes responsive to BMP signaling in mouse dental mesenchymal cells, while the set of genes responsive to BMP signaling in mouse dental epithelial cells was not significantly altered. Perhaps consistent with a relatively lesser effect on dental epithelia than mesenchyme in the *Bmp6* mutant, *Sox2*, implicated in epithelial stem cells during tooth replacement in other polyphyodonts [37–39], was not significantly affected in *Bmp6* mutants.

### Potential parallels between tooth and hair regeneration

In other vertebrates that undergo tooth replacement, dental stem cells have been proposed to mediate tooth replacement [37–39,64–66]. Teeth develop from placodes, transient epithelial thickenings that grow outwards or inwards to form epithelial appendages [42,43]. Teeth are developmentally deeply homologous to other placode-derived organs, such as mammalian hair [44,45,67]. Mammalian hairs, like fish teeth, are constantly replaced throughout adult life. During mammalian hair regeneration, *Bmp6* regulates stem cell quiescence in the hair follicle stem cell niche [40,46]. Additionally, conditional knockout of the BMP receptor *Bmpr1a* in mouse hair follicles resulted in a loss of both hair regeneration and stem cell signature genes [46]. Thus, we hypothesized that stickleback *Bmp6* might regulate similar genetic pathways during tooth replacement as during hair regeneration. Supporting this hypothesis, in *Bmp6* mutant tooth plate tissue, we found a significant downregulation of mouse hair follicle stem cell signature genes, a set of genes previously described to be upregulated in mouse hair follicle stem cells compared to cells in the forming hair germ [46]. This result supports a model where modulating *Bmp6* expression in derived freshwater sticklebacks alters dental stem cell dynamics to result in the elevated tooth replacement rate seen in high-toothed freshwater sticklebacks [26]. Furthermore, this result suggests that the genetic circuitry regulating stem cell quiescence in continuously regenerating mammalian hair may be shared during constant tooth replacement in fish. This shared gene set might reflect an ancient highly conserved pathway regulating vertebrate epithelial appendage regeneration. If so, further identifying this core conserved gene regulatory network would provide profound insights into vertebrate development, regeneration, and evolution.

## Methods

### Ethics statement

All animal experiments (including euthanasia by immersion in a buffered 250 mg/L tricaine methane sulfonate solution) were done with the approval of the Institutional Animal Care and Use Committee from University of California, Berkeley (protocol #R330).

### Stickleback Husbandry

Stickleback fish were raised in 29-gallon tanks in ~1/10^th^ ocean water (3.5 g/l Instant Ocean salt, 0.4 mL/l NaHCO3) and fed live brine shrimp as larvae, then frozen daphnia, bloodworms, and Mysis shrimp as juveniles and adults. All fish crosses were conducted using artificial fertilization.

### Recombinant mapping

Further F3-F5 generations of a Paxton Benthic freshwater by Little Campbell marine F2 cross [68] were propagated by intercrossing fish heterozygous for marine and benthic alleles of chromosome 21 (identified by heterozygosity at Stn487 and Stn489). Recombinant fish in F4- F5 generation were identified using microsatellite markers Stn487 and Stn489 which flank the genetic interval surrounding *Bmp6*. Caudal fin tissue was genotyped by first isolating DNA by incubating for 20’ at 94°C, then digesting with 2.5 μL of 20mg/ml proteinase K in lysis buffer (10mM Tris, pH 8.3; 50 mM KCL; 1.5 mM MgCl_2_; 0.3% Tween-20 0.3% NP-40) for an hour at 55°C followed by 20 minutes at 94°C. One μl of undiluted DNA was used directly in the genotyping PCR. Once recombinant fish were identified, recombinant breakpoints were further mapped using a combination of microsatellite markers and restriction fragment length polymorphisms (RFLPs). Primer sequences for the left and right markers used to refine each recombinant chromosome used in this study are shown in S1 Table. Gene content was determined by hand annotating the Ensembl predicted gene list.

Recombinant fish were crossed to F4-F5 fish heterozygous for marine and benthic chromosome 21 that were also derived from the same F2 grandparents. The recombination events in crosses 1–3 were between markers Stn488 and Stn489 (cross 1), or between markers Stn487 and Stn488 (crosses 2 and 3). Genotypes of chromosome 21 in these three crosses were scored as M (marine), B (benthic), or R (recombinant) based upon the two locus genotypes of Stn488/Stn489 (cross 1) or Stn487/Stn488 (crosses 2 and 3).

Recombinant crosses were raised to ~30 mm standard length. Fish were stained for bone with Alizarin Red, cleared, and pharyngeal teeth were quantified as previously described [19]. If tooth number was significantly correlated with standard fish length, sex, or family, we corrected for each using a linear model and used residuals from that regression for statistical analysis (S1 Table). To test whether each recombinant chromosome contained the tooth number QTL, we performed a likelihood-ratio test comparing two models, one with the recombinant chromosome behaving as a benthic chromosome and one with the recombinant chromosome behaving as a marine chromosome.

### Benthic by marine F2 crosses

Lab-reared stocks of Paxton Benthic fish used for F2 crosses were generated by incrossing wild-derived fish from Paxton Benthic lake, British Columbia. Five benthic fish were crossed to marine fish and F1s subsequently incrossed to generate six F2 crosses. The specifics of marine populations used in each cross are presented in S2 Table. Three microsatellite markers spanning the chromosome 21 tooth QTL were genotyped: CM1440 (primer sequences 5’ to 3’: AAATGTGCTCCTGGATGTGC and CTTTCTCCTTCTGCCAAACG), Stn489, and Stn488; this set of genotypes was used to define molecularly distinct chromosome 21s. F2 crosses 5 and 6 shared a benthic grandparent. This marker analysis suggests that there are eight molecularly distinct chromosome 21s in the five benthic grandparents.

To determine the effect of chromosome 21 on tooth number, the F2 crosses were genotyped using microsatellites markers on chromosome 21 near the tooth QTL (see S2 Table for details). The effects of fish size on tooth number were removed by linear regression and the residuals were back-transformed to the mean standard fish length in each cross. Statistical association between chromosome 21 genotype and back-transformed phenotypes was tested using an ANOVA in R. To determine if both benthic chromosomes had an effect on tooth number in each cross, we performed a likelihood-ratio test for each wild benthic chromosome comparing a model where that chromosome does not have an effect on tooth number to a model where both benthic chromosomes have an equal effect on tooth number.

### Genome sequences of marine and benthic stickleback fish

We resequenced the genomes of the four benthic grandparents from crosses 1–4 and F2 fish homozygous for chromosome B_7_ and B_8_. We also sequenced the marine Little Campbell grandparents from crosses 5–6, and the Japanese marine grandparent from cross 3 (Fig 2). Caudal fins were digested overnight at 55°C in Tail Digestion Buffer (10 mM Tris, pH 8.0, 100 mM NaCl, 10 mM EDTA, pH 8.0, 0.5% SDS, 10 μl of 20mg/ml proteinase K). Genomic DNA was purified with a phenol:choloroform extraction followed by ethanol precipitation. Genomic libraries were generated using the Nextera DNA Sample Prep Kit (Epicentre Biotechologies), the Nextra DNA Sample Preparation Kit (Illumina), or the Nextera XT DNA Library Preparation Kit (Illumina). Paired-end reads (100 bp) were sequenced using an Illumina HiSeq2000. See S3 Table for details of library preparation and sequencing summary for each library.

### Variant calling and tooth specific variant identification

Resulting reads were aligned to the repeat masked verision of the reference stickleback genome [24] using the bwa aln and bwa sampe modules of the burrows-wheeler aligner [69]. As the genome assemblies in the minimal 884 kb meiotic interval are identical in the Jones et al. and Glazer et al. assemblies [24,70], the original Jones et al. assembly was used [24]. Samtools (version 0.1.17) [71] was used to create a sorted and indexed BAM file, and Picard tools (version 1.51) (http://broadinstitute.github.io/picard/) was used to fix mate information, add read groups, and remove PCR duplicates. GATK's Unified Genotyper (parameters: '—genotype_likelihoods_model INDEL', '-stand_call_conf 25', and '-stand_emit_conf 25') RealignerTargetCreator, IndelRealigner (parameter: '-LOD 0.4') was used to call potential target indels and perform realignment around indels. Base quality recalibration was accomplished using BaseRecalibrator. HaplotypeCaller (parameters: '-emitRefConfidence GVCF', '—variant_index_type LINEAR', and '—variant_index_parameter 128000') was used to generating a genomic VCF (gVCF) file for each library. The resulting gVCFs were merged and variants were called using the GenotypeGVCFs module [72–74]. High quality variants were selected using the following criteria: 1) Variants must have a variant quality score greater than 400. 2) Variants must not be called 'missing' or have a quality score of less than 10 in either high-coverage benthic genome. 3) Variants must not be called 'missing' or have a quality score of less than ten in no more than two genomes. To further remove stickleback specific repeats, we removed variants with >99% of the 100bp flanking sequence matching more than six places in the genome using blastn with an e-value of less than 1x10^-30^ [75]. QTL concordance score is the absolute value of the proportion of times a variant was present in benthic fish with a chromosome 21 tooth QTL minus the proportion of times the same variant was found in fish without a tooth QTL. QTL Concordance scores were calculated using a custom python script.

### Generation of transgenic enhancer stickleback lines

To generate GFP reporter constructs, each of the intron 4 fragments from the Little Campbell marine grandparent from cross 5 was cloned upstream of the *Hsp70l* promoter in a Tol2 expression construct using *Nhe*I [33]. For the mCherry construct, we cloned mCherry into the *Hsp70l* reporter construct using *Sal*I and *Cla*I and the inserts were cloned upstream using *Nhe*I and *BamH*I. Primers for construct generation and sequencing are shown in S5 Table.

To generate transgenic stickleback, transposase messenger RNA was synthesized from pCS2-TP [76] plasmid linearized with *Not*I and transcribed using the mMessage SP6 *in vitro* transcription kit (Ambion) and purified using the Qiagen RNeasy column. One-cell marine stickleback embryos were injected with a mixture of 37.6 ng/μL plasmid DNA and 75 ng/μL RNA with 0.05% phenol red as previously described [33]. All transgene images presented are from stable lines except for the mCherry expression in Fig 4I and 4J and the ~2kb fragment in S1 Fig (which were mosaic).

### Generation of TALEN construct targeting stickleback *Bmp6*

To generate a TALEN pair to target the stickleback *Bmp6* gene, we used the TAL effector Nucleotide Targeter 2.0 (https://tale-nt.cac.cornell.edu/node/add/talen)) to scan the second exon sequence of *Bmp6* for potential target sites [77,78]. We chose TALEN parameters as described [34]. We chose a target site that is unique to *Bmp6* in the stickleback genome and contains a common restriction site, *EcoR*I, which can be used to detect molecular deletions. We assembled the two TALEN constructs using Golden Gate cloning into the destination vectors pCS2TALDD and pCS2TALRR and verified correct assembly using Sanger sequencing as described [34]. See S6 Table for the specifics of the *Bmp6* TALEN design.

### Synthesis and injection of TALEN RNA into stickleback embryos

5’-capped mRNA for each TALEN pair was transcribed using the SP6 mMessage Machine (Ambion) after the TALEN plasmid templates had been linearized with *Not*I. Pooled TALEN mRNA was injected into one-cell PAXB freshwater benthic stickleback embryos at a concentration of 40 ng/μL for each mRNA with 0.05% phenol red.

### Talen mutation identification

To genotype fish for TALEN induced mutations, DNA was extracted as described above from adult fish caudal fin tissue or homogenized whole 1–3 dpf embryos. Genotyping PCR was performed using forward primer 5’- ACAAGCCGCTAAAAAGGACA-3’ and reverse primer 5’- GCACGTGTGCATGCTTTAGA -3’. The reaction profile for the NEB Phusion reaction was 98°C for 30 seconds, 39 cycles of 98°C for 10 seconds, 58°C for 15 seconds, 72°C for 30 seconds, followed by 72°C for 10 minutes. The PCR products were cut directly with *EcoR*I. The products from the wild-type and mutant alleles are cut and not cut, respectively, by this assay (See S3 Fig).

### Tooth patterning quantification

Dorsal and ventral pharyngeal tooth number was quantified on a DM2500 Leica microscope using a TX2 filter as previously described [19]. For both ventral and dorsal tooth counts, total tooth number equals the sum of the left and right sides (of ventral and dorsal pharyngeal teeth, respectively). Tooth plate area and spacing of the ventral pharyngeal tooth plate were quantified from a gray scale image taken with a DFC340 FX camera on a Leica M165FC as previously described [19]. Area and spacing of the ventral pharyngeal tooth plates are the averages of the left and right tooth plate. Skeletal traits were binned by total fish length for three stages: early juvenile <27 mm, late juvenile 27–37 mm, and adults >37 mm.

### RNA purification, sequencing, and alignment

Ventral tooth plates from three wild-type and homozygous mutant (for the 13 bp deletion allele) *Bmp6* female sticklebacks (standard length ~25 mm) were dissected, placed into TRI reagent (Sigma-Aldrich) on ice, ground with a disposable pestle, and frozen overnight at -80°C. The next day, RNA was extracted, isopropanol precipitated, and resuspended in DEPC-treated water. 200 ng of purified RNA was used with Illumina's Truseq Stranded mRNA Library Prep Kit to create sequencing libraries. The resulting bar-coded libraries were pooled and 100 bp paired end reads were generated using a single lane of an Illumina HiSeq2000. Reads were mapped to the stickleback reference genome [24] using STAR (parameters: '—alignIntronMax 200000' '—alignMatesGapMax 200000' '—outFilterMultimapNmax 8') [79]. BAM files were created, sorted, and indexed using Samtools (version 0.1.17)[71]. Picard tools (version 1.51) was used to fix mate information, add read groups, and remove PCR duplicates (http://broadinstitute.github.io/picard/). Using the Ensembl reference transcriptome [24], transcripts were quantified using cuffquant version 2.2.1 (parameters: '-u' '—library-type fr-firststrand') and normalized using cuffnorm [80,81]. Principal component analysis of the resulting transcript abundances was done using the PCA package of FactoMineR (http://factominer.free.fr/index.html) in R, and was plotted in R. GO term enrichment for genes ranked by expression correlation with the first principal component of the RNAseq expression matrix was performed using GOrilla [82,83]. Hierarchical clustering was done using Cluster3.0 (parameters: '-l' '-cg a' '-g 2' '-e 0' '-m c') [84], and the results were visualized using JavaTreeView (version 1.1.6r4)[85]. Additional figures and analyses were done using custom python scripts and figures created using matplotlib.

### Gene set enrichment analysis

ToothCODE gene sets were downloaded from the ToothCODE database (http://compbio.med.harvard.edu/ToothCODE/). ToothCODE identified downstream targets of Bmp signaling by literature mining manipulations of *Bmp2*, *Bmp4*, and *Bmp7*. Targets that were upregulated when BMP signaling increased or downregulated when BMP signaling was decreased were termed BMP target genes. Stickleback orthologs of mouse hair follicle stem cell signature genes, genes upregulated in the hair follicle bulge relative to the hair germ [46] were identified using Ensembl predictions. Statistical enrichment was done similar to the methods as previously described [86]. Each gene in a set was subject to a t-test, obtaining a list of z-scores. The null hypothesis, that the gene set displays no differential expression enrichment, (i.e. t-test z-scores are drawn from a standard normal distribution) was tested using a 1-sample t-test, with resulting *P* values subject to a Bonferroni correction. The significance cutoff for the 1-sample t-test was confirmed by creating a simulated null distribution, using 10,000 permutations of an equal number of genes as in each gene set, randomly chosen without replacement. Cutoff test statistic values were chosen by taking the values at the 100-(2.5/N) and 2.5/N percentile in the simulated null distribution, where N was the number of hypotheses being tested. Analysis was done using a set of custom python scripts, available upon request.

## Supporting information

S1 Fig ~2 kb intron 4 region is an enhancer active in developing fins and teeth. (A) The marine ~2 kb intronic enhancer drove expression at 8 dpf in the distal edges of the developing median fin (arrow) and pectoral fin (arrowhead). (B-C) By 10 dpf, the enhancer drove GFP expression in tooth mesenchyme (arrow) and diffusely in the tooth epithelium (arrowheads) in pharyngeal (B) jaws. GFP expression was also detected in developing tooth germs (arrow) in the oral (C) jaws. In B-C, bone is counterstained with red fluorescence by Alizarin Red. B is a dorsal view of the dissected ventral pharyngeal jaw, while C is a lateral view with anterior to the left of the upper jaw (premaxilla, top) and lower jaw (dentary, bottom). (D) This ~2 kb enhancer controlled dynamic expression throughout development, becoming more restricted to the mesenchyme as the tooth matures. Scale bars are 100 μm (A-C) and 50 μm (D).
(TIF)

S2 Fig Efficacy of *Bmp6* TALENs in stickleback embryos. (A) Frequencies of wild-type (+/+), heterozygous (+/-), and homozygous (-/-) mutant F_0_-injected 3 days post fertilization (dpf) embryos are shown for three independent injection rounds. (B) An *EcoR*I site was destroyed by induced mutations. Representative *EcoR*I digest assays on PCR amplicon from genomic DNA from a homozygous wild-type (left, +/+), heterozygous (middle, +/-), and homozygous mutant (right, -/) injected embryo are shown.
(TIF)

S3 Fig Predicted amino acid alignments of the wild-type, 13bp deletion, and the 3 bp deletion/4 bp insertion alleles of BMP6. Predicted mutant BMP6 sequences, 3bp deletion/4bp insertion (middle) and 13bp deletion (bottom), aligned to wild-type (top) BMP6 sequence. The 13bp deletion and the 3bp deletion + 4bp insertion generate frameshifts that result in premature stop codons (marked by asterisk) in the 2^nd^ and 3^rd^ exons, respectively, predicted to truncate the protein. Wild-type BMP6 sequences and intron/exon boundaries (marked with arrowheads) were previously described [19]. The position of the *EcoR*I site used as the genotyping assay is noted.
(TIF)

S4 Fig *Bmp6* mutation effects on dorsal pharyngeal teeth. (A) Size-corrected pharyngeal tooth number on dorsal tooth plate 1 (DTP1) were not significantly different between homozygous mutant (red), heterozygous (purple), and homozygous wild-type (blue) fish at any stage. (B) The dorsal tooth plate 2 (DTP2) tooth numbers were only significant at the adult stage (ANOVA *P* = 0.028) in contrast to the ventral pharyngeal teeth (VTP) results (see Fig 5).
(TIF)

S1 Table Summary of recombinant crosses. Sample sizes of the Paxton benthic x Little Campbell marine recombinant crosses are shown along with the primer sequences for left and right genotyping markers used as the boundaries for the recombination breakpoint. The markers for recombinant 1 and 2 are size polymorphisms and the markers for recombinant 3 are restriction fragment length polymorphisms (RFLPs) using the restriction nuclease shown. Standard fish length and sex were corrected for when appropriate and corrections performed for each cross are listed. For each left and right marker, the left and right positions in base pairs, respectively, on chromosome 21 in the stickleback genome assembly [24] are listed.
(PDF)

S2 Table Benthic x marine F2 cross summary. Results from marine by benthic F2 crosses testing eight benthic chromosomes. Populations, number of F2 fish, and chromosome 21 marker genotyped for each cross are listed. PAXB = Paxton benthic, JAMA = Japanese marine, RABS = Rabbit Slough marine, LITC = Little Campbell river marine. Sex of each grandparent is indicated in “Populations crossed (male x female)” column. For each cross, the most informative and completely genotyped marker nearest to the previously reported QTL peak [19] is listed. Standard length effects on total ventral pharyngeal tooth number were corrected for when appropriate and residuals were back-transformed to the mean standard fish length within each cross. Mean and standard error of corrected tooth number are shown for marine homozygotes (MM), heterozygotes (MB), and benthic homozygotes (BB). All PAXB grandparents were different fish, except the grandparent of crosses 5 and 6, which was the same PAXB male fish. The eight different molecularly distinct benthic chromosomes (see Fig 2) are listed in “Benthic chromosomes tested” column. Crosses 1, 3, and 4 tested two distinct benthic chromosomes and crosses 2, 5, and 6 tested a single benthic chromosome. Crosses 1 and 4 share a benthic chromosome with the same microsatellite genotypes (see Methods). *P* values from ANOVAs for testing whether genotype significantly effects tooth number phenotype are listed (see Fig 2). The last column shows *P* values from two likelihood ratio (LR) tests comparing the additive model to no effect benthic 1 model and no effect benthic 2 model are shown. The four allele marker used for crosses 1, 3, and 4 were CM1440, Stn223, and CM1440, respectively. The LR tests show that both benthic chromosomes have significant effects on tooth number in crosses 1,3, and 4. F2 crosses 2, 5, and 6 contain the same benthic chromosome and thus, in these crosses the benthic chromosomes can not be tested individually (since they can not be molecularly distinguished). So for these crosses, the LR test is not applicable (NA).
(PDF)

S3 Table Summary of genome resequencing. For each fish used for genome resequencing, library preparation kit, total reads, final mapped reads, and estimated coverage are listed. LITC, JAMA, and PAXB refer to the Little Campbell Marine, Japanese Marine, and Paxton Benthic populations, respectively. Estimated coverage was calculated by dividing the final mapped reads by the stickleback genome size for each sample. The two high coverage genomes (>70 x) were each sequenced in a full lane and the lower coverage genomes were barcoded and multiplexed with five other fish per sequencing lane. All sequencing was 100 bp paired-end on Illumina HiSeq2000.
(PDF)

S4 Table QTL-associated variants. “Chr. 21 position” indicates position on chromosome 21 in stickleback reference genome assembly. “Reference” lists genotype at that position in reference genome assembly [24], while “QTL-associated variant” indicates genotype at that position of variants concordant with presence or absence of tooth QTL (see Fig 3).
(PDF)

S5 Table Reporter construct cloning primers. Sequences of forward and reverse primers used to clone each construct are listed 5’ to 3’ along with the restriction enzyme used to digest the PCR amplicon. The orientation of the inserts in the GFP constructs were tested in the minus direction relative to the promoter since the endogenous enhancer is 3’ to the *Bmp6* promoter in the stickleback genome. The mCherry construct was cloned in the plus orientation to mirror the orientation for the 5’ tooth enhancer transgenic line used in the co-labeling experiment (see Fig 4).
(PDF)

S6 Table Custom TALEN design and targets. Repeat Variable Diresidues **(**RVDs) used to generate left (TAL1) and right (TAL2) nuclease pairs targeting the second exon of *Bmp6*, and stickleback target sequence is listed. Underlined nucleotides correspond to the 19bp TAL1 and TAL2 targets flanked by the 17bp spacer containing an *EcoR*I restriction site (bold).
(PDF)

S7 Table *Bmp6* mutant class survival and fish length. Sample sizes, mean total fish lengths, and standard deviations for crosses generating wild-type, heterozygous, and homozygous mutant fish (intercrosses, top) or wild-type and heterozygotes (backcrosses, bottom) are shown. Mortality *P* values from a Chi-square test expecting a 1:2:1 ratio for the intercrosses and a 1:1 ratio for the backcrosses are shown. There was significant deviation from expected 1:2:1 ratios (likely due to mortality) in intercross clutch C, where the fish were the largest. Length *P* values from an ANOVA are shown for a recessive model (Wild-type and heterozygous classes are merged and compared to the homozygous mutants). In all three intercrosses, homozygous mutant fish were smaller than their heterozygous and wild-type siblings. One of the backcross clutches had a significant size defect, which was not seen in the other clutch. Crosses A, C, D, and E contain the 13 bp deletion allele. Cross B is a transheterozygous cross between a fish heterozygous for the 13 bp deletion and a fish heterozygous for the 3bp deletion+4bp insertion (see S8 Table).
(PDF)

S8 Table Transheterozygous effects on tooth patterning. Analysis of a transheterozygous cross, 13bp deletion by the 3bp deletion/4bp insertion, for tooth patterning phenotypes. The effect of fish standard length was removed using a linear regression for each meristic or continuous trait. There were significant recessive differences in the homozygous mutant class for ventral tooth number and ventral tooth plate area, consistent with the results of the mutant time course. Continuous trait means and standard deviations (shown in brackets) for each genotypic class along with *P* values from a Tukey post-hoc test are shown.
(PDF)

S9 Table RNA sequencing summary statistics. Total reads, mapped reads and fish standard length are listed for each wild-type (1–3) and mutant fish (4–6) used for sequencing. All libraries were made with the TruSeq Stranded mRNA Library Prep Kit, barcoded, multiplexed and 100 bp paired-end sequenced in a single lane of an Illumina HiSeq2000.
(PDF)

S10 Table GO term enrichment of the first principal component of gene expression. GO term enrichment for a list of genes ranked by expression correlation with the first principal component of the *Bmp6* wild-type and mutant tooth plate expression matrix. Shown are significant GO terms with an FDR q-value less than 1E-04.
(PDF)

S1 File Source data file. Source data for all figures.
(XLSX)
