# Supplementary figures and images for: An intronic enhancer of Bmp6 underlies evolved tooth gain in sticklebacks
Source: PLoS Genet. 2018 Jun 14;14(6):e1007449. doi: 10.1371/journal.pgen.1007449 (PMC6019817; doi:10.1371/journal.pgen.1007449)

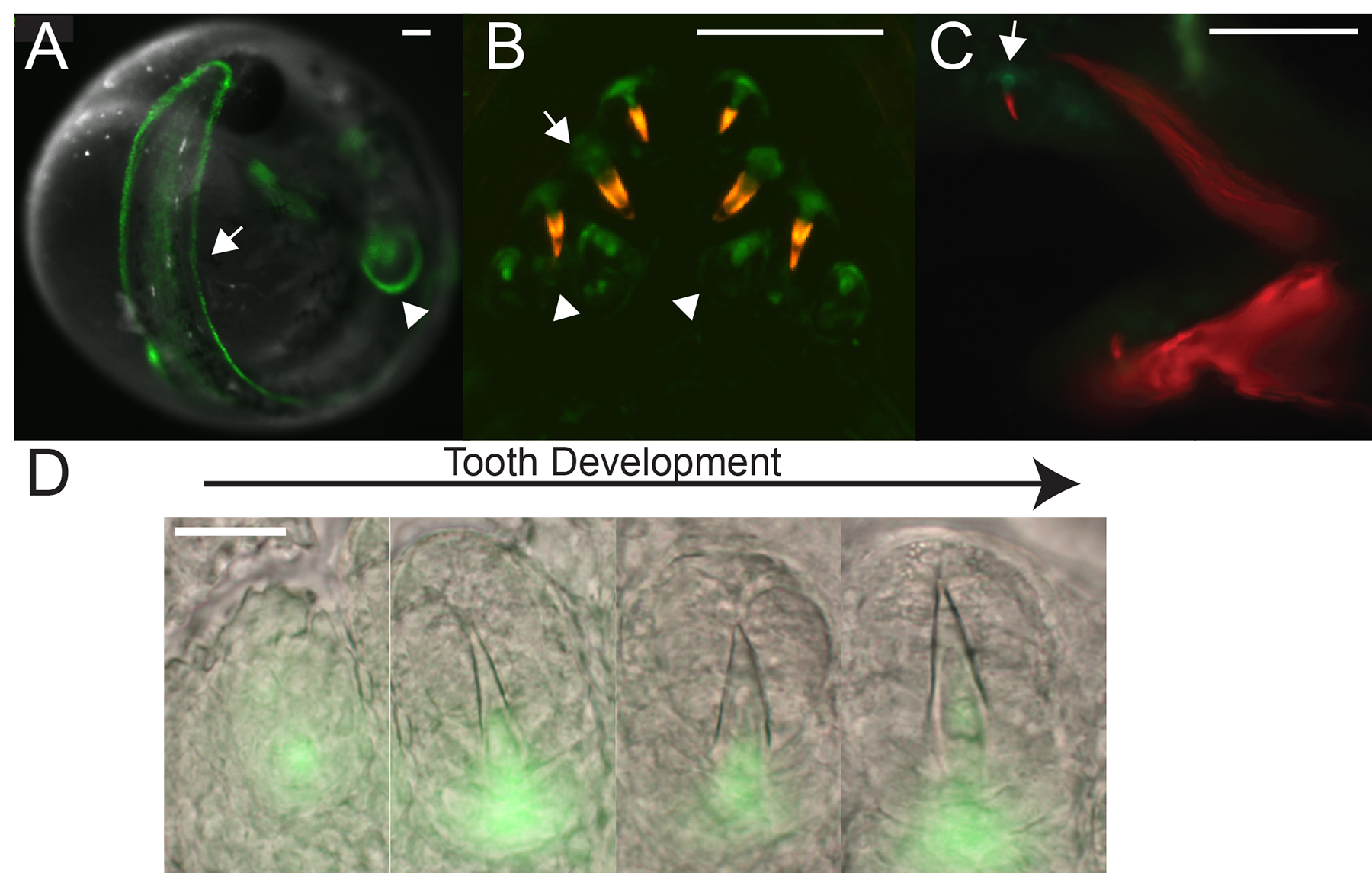

Supplement: S1 Fig — (A) The marine ~2 kb intronic enhancer drove expression at 8 dpf in the distal edges of the developing median fin (arrow) and pectoral fin (arrowhead). (B-C) By 10 dpf, the enhancer drove GFP expression in tooth mesenchyme (arrow) and diffusely in the tooth epithelium (arrowheads) in pharyngeal (B) jaws. GFP expression was also detected in developing tooth germs (arrow) in the oral (C) jaws. In B-C, bone is counterstained with red fluorescence by Alizarin Red. B is a dorsal view of the dissected ventral pharyngeal jaw, while C is a lateral view with anterior to the left of the upper jaw (premaxilla, top) and lower jaw (dentary, bottom). (D) This ~2 kb enhancer controlled dynamic expression throughout development, becoming more restricted to the mesenchyme as the tooth matures. Scale bars are 100 μm (A-C) and 50 μm (D). (TIF) [file pgen.1007449.s001.tif]

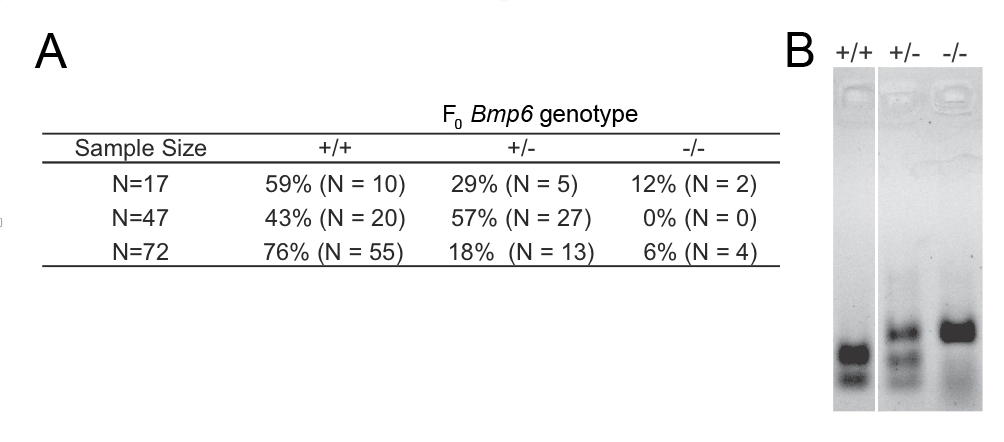

Supplement: S2 Fig — (A) Frequencies of wild-type (+/+), heterozygous (+/-), and homozygous (-/-) mutant F0-injected 3 days post fertilization (dpf) embryos are shown for three independent injection rounds. (B) An EcoRI site was destroyed by induced mutations. Representative EcoRI digest assays on PCR amplicon from genomic DNA from a homozygous wild-type (left, +/+), heterozygous (middle, +/-), and homozygous mutant (right, -/) injected embryo are shown. (TIF) [file pgen.1007449.s002.tif]

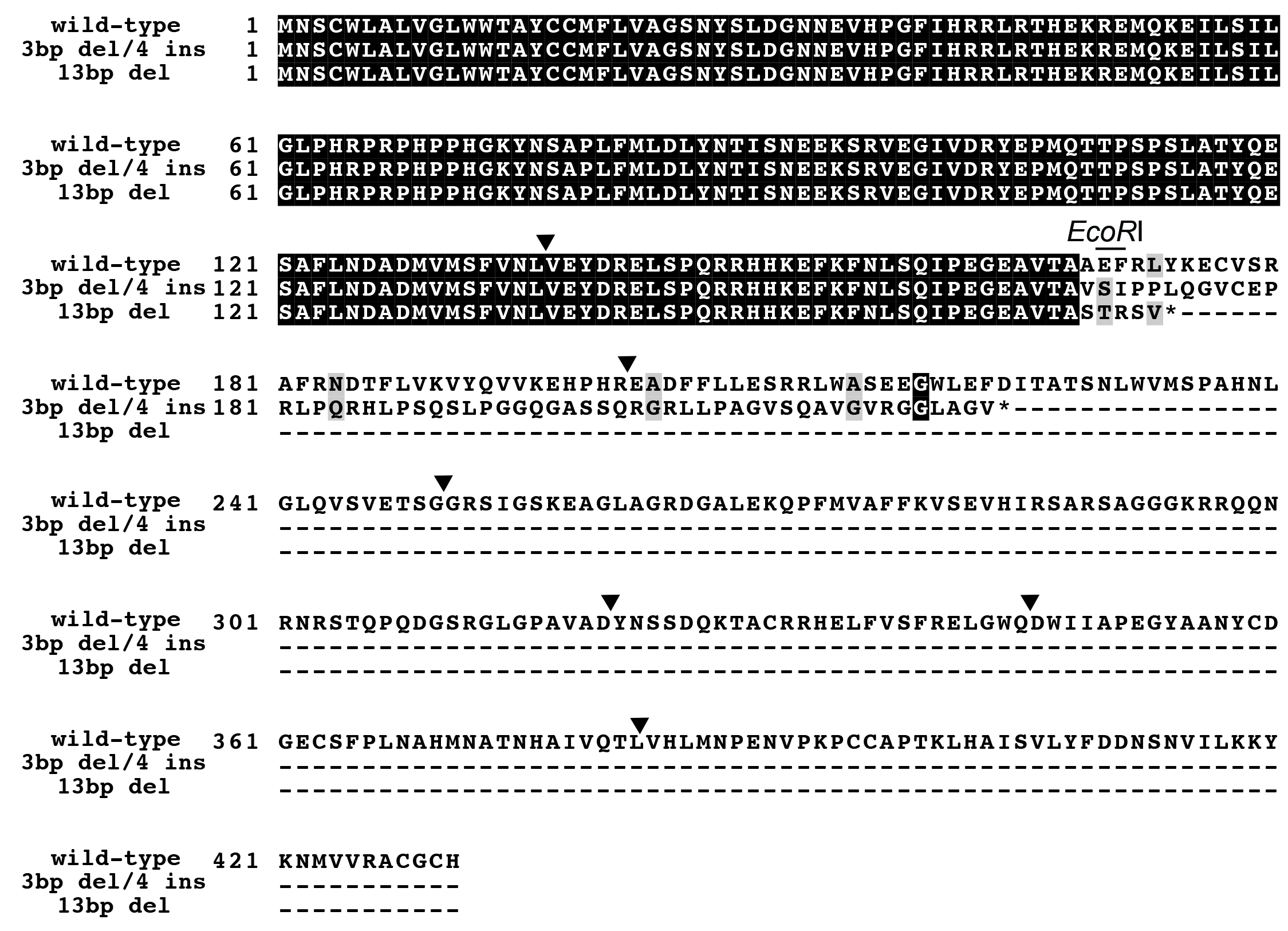

Supplement: S3 Fig — Predicted mutant BMP6 sequences, 3bp deletion/4bp insertion (middle) and 13bp deletion (bottom), aligned to wild-type (top) BMP6 sequence. The 13bp deletion and the 3bp deletion + 4bp insertion generate frameshifts that result in premature stop codons (marked by asterisk) in the 2nd and 3rd exons, respectively, predicted to truncate the protein. Wild-type BMP6 sequences and intron/exon boundaries (marked with arrowheads) were previously described [19]. The position of the EcoRI site used as the genotyping assay is noted. (TIF) [file pgen.1007449.s003.tif]

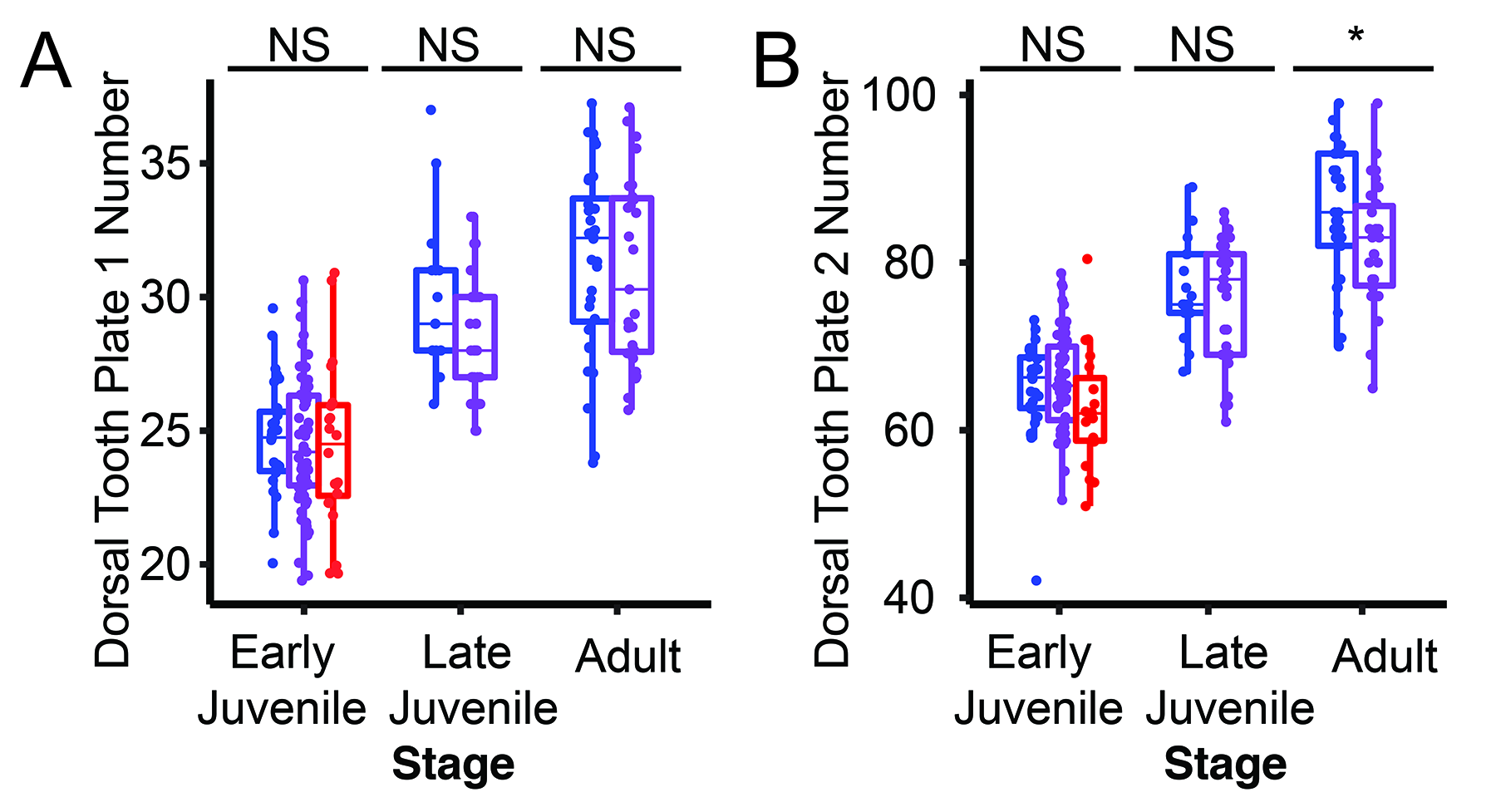

Supplement: S4 Fig — (A) Size-corrected pharyngeal tooth number on dorsal tooth plate 1 (DTP1) were not significantly different between homozygous mutant (red), heterozygous (purple), and homozygous wild-type (blue) fish at any stage. (B) The dorsal tooth plate 2 (DTP2) tooth numbers were only significant at the adult stage (ANOVA P = 0.028) in contrast to the ventral pharyngeal teeth (VTP) results (see Fig 5). (TIF) [file pgen.1007449.s004.tif]
