# Supplementary material for: An intronic enhancer of Bmp6 underlies evolved tooth gain in sticklebacks
Source: PLoS Genet. 2018 Jun 14;14(6):e1007449. doi: 10.1371/journal.pgen.1007449 (PMC6019817; doi:10.1371/journal.pgen.1007449)
Supplement: S1 Table — Sample sizes of the Paxton benthic x Little Campbell marine recombinant crosses are shown along with the primer sequences for left and right genotyping markers used as the boundaries for the recombination breakpoint. The markers for recombinant 1 and 2 are size polymorphisms and the markers for recombinant 3 are restriction fragment length polymorphisms (RFLPs) using the restriction nuclease shown. Standard fish length and sex were corrected for when appropriate and corrections performed for each cross are listed. For each left and right marker, the left and right positions in base pairs, respectively, on chromosome 21 in the stickleback genome assembly [24] are listed. (PDF) [file pgen.1007449.s005.pdf]

Cross	Number of fish	Correction	Primer sequences for recombinant left marker (5’ to 3’); genomic position	Primer sequences for recombinant right marker (5’ to 3’); genomic position	Marker type
1	109	Sex	CACTGAAGCCGGAGGAGAGG, ATCAGAGAGGGTCCAGAACG; 3881415	AGTCCGCCACTTGTCTTTCC, GTCATGCAGACCATGATTCC; 3909782	Size polymorphism
2	147	Fish length and sex	TGAACCAATTGTTTGGAACATC, AATCGCCATGTCAAATTCCT; 2896862	CCCGCAAGAAAGCAATTTAT, TTTGTTTCCTGCCTTCGAGT; 2918674	Size polymorphism
3	102	Fish length and sex	ATCCAGCCCAGAGTGAAATG, GGCCTACCAACTTGACCGTA; 3026115	TGTGTGCAAACACACAGCAT, TCTGCTCTGCTTTGCTTCTTC; 3093809	RFLP (*Ava*II left and *Sal*I right)
