# Supplementary material for: An intronic enhancer of Bmp6 underlies evolved tooth gain in sticklebacks
Source: PLoS Genet. 2018 Jun 14;14(6):e1007449. doi: 10.1371/journal.pgen.1007449 (PMC6019817; doi:10.1371/journal.pgen.1007449)
Supplement: S2 Table — Results from marine by benthic F2 crosses testing eight benthic chromosomes. Populations, number of F2 fish, and chromosome 21 marker genotyped for each cross are listed. PAXB = Paxton benthic, JAMA = Japanese marine, RABS = Rabbit Slough marine, LITC = Little Campbell river marine. Sex of each grandparent is indicated in “Populations crossed (male x female)” column. For each cross, the most informative and completely genotyped marker nearest to the previously reported QTL peak [19] is listed. Standard length effects on total ventral pharyngeal tooth number were corrected for when appropriate and residuals were back-transformed to the mean standard fish length within each cross. Mean and standard error of corrected tooth number are shown for marine homozygotes (MM), heterozygotes (MB), and benthic homozygotes (BB). All PAXB grandparents were different fish, except the grandparent of crosses 5 and 6, which was the same PAXB male fish. The eight different molecularly distinct benthic chromosomes (see Fig 2) are listed in “Benthic chromosomes tested” column. Crosses 1, 3, and 4 tested two distinct benthic chromosomes and crosses 2, 5, and 6 tested a single benthic chromosome. Crosses 1 and 4 share a benthic chromosome with the same microsatellite genotypes (see Methods). P values from ANOVAs for testing whether genotype significantly effects tooth number phenotype are listed (see Fig 2). The last column shows P values from two likelihood ratio (LR) tests comparing the additive model to no effect benthic 1 model and no effect benthic 2 model are shown. The four allele marker used for crosses 1, 3, and 4 were CM1440, Stn223, and CM1440, respectively. The LR tests show that both benthic chromosomes have significant effects on tooth number in crosses 1,3, and 4. F2 crosses 2, 5, and 6 contain the same benthic chromosome and thus, in these crosses the benthic chromosomes can not be tested individually (since they can not be molecularly distinguished). So for these crosses, [file pgen.1007449.s006.pdf]

**Cross**	**Populations crossed (male x female)**	**# of F2s**	**Marker**	**MM**	**MB**	**BB**	**Benthic chromosomes tested**	**Correction**	**ANOVA *P*-values**	**Likelihood ratio test *P*-values**
**1**	PAXB x JAMA	92	CM1440	71 (1.2)	75 (1.6)	80 (1.3)	B_1 and B_2	Fish Length	**0.002**	**0.01, 0.03**
**2**	PAXB x JAMA	51	CM1440	61 (1.6)	63 (1.6)	68 (1.9)	B_3	Fish Length	**0.024**	NA
**3**	PAXB x RABS	62	Stn489	77 (3.1)	82 (2.1)	96 (2.4)	B_4 and B_5	None	**0.0005**	**0.0005, 0.02**
**4**	JAMA x PAXB	77	Stn489	72 (1.7)	73 (2)	81 (2.9)	B_1 and B_6	Fish Length	**0.004**	**0.03, 0.04**
**5**	PAXB x LITC	138	Stn487	62 (1.4)	65 (0.9)	72 (1.5)	B_7	None	**2.11×10^{-5}**	NA
**6**	PAXB x LITC	75	Stn487	72 (3.1)	74 (1.9)	74 (2.7)	B_8	Fish Length	0.69	NA
