# Supplementary material for: An intronic enhancer of Bmp6 underlies evolved tooth gain in sticklebacks
Source: PLoS Genet. 2018 Jun 14;14(6):e1007449. doi: 10.1371/journal.pgen.1007449 (PMC6019817; doi:10.1371/journal.pgen.1007449)
Supplement: S3 Table — For each fish used for genome resequencing, library preparation kit, total reads, final mapped reads, and estimated coverage are listed. LITC, JAMA, and PAXB refer to the Little Campbell Marine, Japanese Marine, and Paxton Benthic populations, respectively. Estimated coverage was calculated by dividing the final mapped reads by the stickleback genome size for each sample. The two high coverage genomes (>70 x) were each sequenced in a full lane and the lower coverage genomes were barcoded and multiplexed with five other fish per sequencing lane. All sequencing was 100 bp paired-end on Illumina HiSeq2000. (PDF) [file pgen.1007449.s007.pdf]

Fish	Library Preparation Kit	Total Reads	Final Mapped Reads	Est. Coverage
Cross 1 - PAXB Grandparent	NEXTERA XT DNA sample preparation kit	45084360	39022926	8
Cross 2 - PAXB Grandparent	NEXTERA XT DNA sample preparation kit	39144478	33907774	7
Cross 2 – JAMA Grandparent	NEXTERA DNA sample preparation kit	59900872	43098831	9
Cross 3 - PAXB Grandparent	NEXTERA XT DNA sample preparation kit	42228302	26568000	6
Cross 4 - PAXB Grandparent	NEXTERA XT DNA sample preparation kit	38992384	32095135	7
Cross 5 – PAXB Homozygous F2	Epicenter NEXTERA	482809124	323700364	70
Cross 5 - LITC Grandparent	NEXTERA DNA sample preparation kit	70365294	58516503	13
Cross 6 – LITC Grandparent	NEXTERA DNA sample preparation kit	69017068	58385657	13
Cross 6 – PAXB Homozygous F2	Epicenter NEXTERA	450163966	326212116	71
