# Supplementary material for: An intronic enhancer of Bmp6 underlies evolved tooth gain in sticklebacks
Source: PLoS Genet. 2018 Jun 14;14(6):e1007449. doi: 10.1371/journal.pgen.1007449 (PMC6019817; doi:10.1371/journal.pgen.1007449)
Supplement: S4 Table — “Chr. 21 position” indicates position on chromosome 21 in stickleback reference genome assembly. “Reference” lists genotype at that position in reference genome assembly [24], while “QTL-associated variant” indicates genotype at that position of variants concordant with presence or absence of tooth QTL (see Fig 3). (PDF) [file pgen.1007449.s008.pdf]

Chr. 21 position	Reference	QTL-associated variant
3853687	G	C
3855372	A	C
3856007	T	A
3856021	C	T
3856164	G	A
3856390	T	A
3856434	G	T
3856444	A	G
3857276	C	T
3858044	C	T
