# Supplementary material for: An intronic enhancer of Bmp6 underlies evolved tooth gain in sticklebacks
Source: PLoS Genet. 2018 Jun 14;14(6):e1007449. doi: 10.1371/journal.pgen.1007449 (PMC6019817; doi:10.1371/journal.pgen.1007449)
Supplement: S5 Table — Sequences of forward and reverse primers used to clone each construct are listed 5’ to 3’ along with the restriction enzyme used to digest the PCR amplicon. The orientation of the inserts in the GFP constructs were tested in the minus direction relative to the promoter since the endogenous enhancer is 3’ to the Bmp6 promoter in the stickleback genome. The mCherry construct was cloned in the plus orientation to mirror the orientation for the 5’ tooth enhancer transgenic line used in the co-labeling experiment (see Fig 4). (PDF) [file pgen.1007449.s009.pdf]

Construct	Forward primer (5’ to 3’)	Reverse primer (5’ to 3’)	Enzyme	Orientation
~2 kb enhancer GFP	GCCGGCTAGCACCGACACAGCTGTACTTGG	GCCGGCTAGCAGAGTCCTGATGGCCTCTCC	*Nhe*I	Minus
~1.3 kb enhancer GFP	GCCGGCTAGCGAGAGCATCCGTCTTGTGGG	GCCGGCTAGCAGAGTCCTGATGGCCTCTCC	*Nhe*I	Minus
511 bp enhancer GFP	GCCGGCTAGCGTGTGTGCGCGGTGGAAAATG	GCCGGCTAGCAGAGTCCTGATGGCCTCTCC	*Nhe*I	Minus
511 bp enhancer mCherry	GCCGGCTAGCGTGTGTGCGCGGTGGAAAATG	GCCGGGATCCAGAGTCCTGATGGCCTCTCC	*Nhe*I and *BamH*I	Plus
