# Supplementary material for: An intronic enhancer of Bmp6 underlies evolved tooth gain in sticklebacks
Source: PLoS Genet. 2018 Jun 14;14(6):e1007449. doi: 10.1371/journal.pgen.1007449 (PMC6019817; doi:10.1371/journal.pgen.1007449)
Supplement: S6 Table — Repeat Variable Diresidues (RVDs) used to generate left (TAL1) and right (TAL2) nuclease pairs targeting the second exon of Bmp6, and stickleback target sequence is listed. Underlined nucleotides correspond to the 19bp TAL1 and TAL2 targets flanked by the 17bp spacer containing an EcoRI restriction site (bold). (PDF) [file pgen.1007449.s010.pdf]

Target	TAL1 (left), TAL2 (right) RVDs	TAL1,TAL2 Lengths	Spacer	Target Stickleback Sequence (5' to 3')
Bmp6 exon 2	TAL1: HD HD HD NI NN NI NN NN NN HD NN NI NN NN HD HD NN NG HD<br>TAL2: HD NI HD NI HD NI HD NG HD HD HG HG NN NG NI NN NI NN NN	19bp	17 bp	CCCAGAGGGCGAGGCCGTC accgcagca**gaattc**cg CCTCTACAAGGAGTGTGTG
