# Supplementary material for: An intronic enhancer of Bmp6 underlies evolved tooth gain in sticklebacks
Source: PLoS Genet. 2018 Jun 14;14(6):e1007449. doi: 10.1371/journal.pgen.1007449 (PMC6019817; doi:10.1371/journal.pgen.1007449)
Supplement: S7 Table — Sample sizes, mean total fish lengths, and standard deviations for crosses generating wild-type, heterozygous, and homozygous mutant fish (intercrosses, top) or wild-type and heterozygotes (backcrosses, bottom) are shown. Mortality P values from a Chi-square test expecting a 1:2:1 ratio for the intercrosses and a 1:1 ratio for the backcrosses are shown. There was significant deviation from expected 1:2:1 ratios (likely due to mortality) in intercross clutch C, where the fish were the largest. Length P values from an ANOVA are shown for a recessive model (Wild-type and heterozygous classes are merged and compared to the homozygous mutants). In all three intercrosses, homozygous mutant fish were smaller than their heterozygous and wild-type siblings. One of the backcross clutches had a significant size defect, which was not seen in the other clutch. Crosses A, C, D, and E contain the 13 bp deletion allele. Cross B is a transheterozygous cross between a fish heterozygous for the 13 bp deletion and a fish heterozygous for the 3bp deletion+4bp insertion (see S8 Table). (PDF) [file pgen.1007449.s011.pdf]

| Intercross | Genotype | Sample size | Mortality *P* value | Fish length | Standard deviation | Length *P* value |
|---|---|---|---|---|---|---|
| **A** | +/+ | 6 | 0.75 | 14.53 | 0.80 | **0.04** |
| | +/- | 16 | - | 14.59 | 0.55 | - |
| | -/- | 6 | - | 13.95 | 0.72 | - |
| **B** | +/+ | 9 | 0.31 | 20.56 | 1.31 | **0.04** |
| | +/- | 27 | - | 20.26 | 1.44 | - |
| | -/- | 8 | - | 19.21 | 1.20 | - |
| **C** | +/+ | 25 | **0.005** | 25.47 | 4.63 | **0.03** |
| | +/- | 55 | - | 25.85 | 4.71 | - |
| | -/- | 9 | - | 22.15 | 2.60 | - |
| Backcross | | | | | | |
| **D** | +/+ | 32 | 0.35 | 37.61 | 3.07 | 0.52 |
| | +/- | 25 | - | 38.21 | 3.77 | - |
| **E** | +/+ | 18 | 0.87 | 41.34 | 2.25 | **0.05** |
| | +/- | 17 | - | 39.38 | 3.15 | - |
