# Supplementary material for: An intronic enhancer of Bmp6 underlies evolved tooth gain in sticklebacks
Source: PLoS Genet. 2018 Jun 14;14(6):e1007449. doi: 10.1371/journal.pgen.1007449 (PMC6019817; doi:10.1371/journal.pgen.1007449)
Supplement: S8 Table — Analysis of a transheterozygous cross, 13bp deletion by the 3bp deletion/4bp insertion, for tooth patterning phenotypes. The effect of fish standard length was removed using a linear regression for each meristic or continuous trait. There were significant recessive differences in the homozygous mutant class for ventral tooth number and ventral tooth plate area, consistent with the results of the mutant time course. Continuous trait means and standard deviations (shown in brackets) for each genotypic class along with P values from a Tukey post-hoc test are shown. (PDF) [file pgen.1007449.s012.pdf]

| **Meristic or Continuous Traits** | **Homozygous wild-type (WW, n = 9)** | **Heterozygous (WM, n = 27)** | **Homozygous mutant (MM, n = 8)** | **WW-WM** | **WM-MM** | **WW-MM** |
|---|---|---|---|---|---|---|
| Ventral Tooth Number | 54.83 (3.34) | 54.46 (2.72) | 49.73 (4.67) | 0.9537 | **0.0024** | **0.0070** |
| Dorsal Tooth Plate 1 Number | 24.35 (2.64) | 25.4 (1.99) | 24.14 (2.6) | 0.4509 | 0.3548 | 0.9804 |
| Dorsal Tooth Plate 2 Number | 66.57 (5.2) | 67.5 (5.45) | 64.47 (6.07) | 0.9023 | 0.3777 | 0.7143 |
| Ventral Tooth Plate Area | 0.08 (0.01) | 0.08 (0.01) | 0.07 (0.01) | 0.6802 | **0.0260** | **0.0157** |
| Average Tooth Spacing | 0.06 (0) | 0.06 (0) | 0.06 (0) | 0.8967 | 0.6829 | 0.9384 |
