# Supplementary material for: An intronic enhancer of Bmp6 underlies evolved tooth gain in sticklebacks
Source: PLoS Genet. 2018 Jun 14;14(6):e1007449. doi: 10.1371/journal.pgen.1007449 (PMC6019817; doi:10.1371/journal.pgen.1007449)
Supplement: S9 Table — Total reads, mapped reads and fish standard length are listed for each wild-type (1–3) and mutant fish (4–6) used for sequencing. All libraries were made with the TruSeq Stranded mRNA Library Prep Kit, barcoded, multiplexed and 100 bp paired-end sequenced in a single lane of an Illumina HiSeq2000. (PDF) [file pgen.1007449.s013.pdf]

Fish	Total Reads	Final Mapped Reads	SL (mm)
Bmp6 +/+ 1	49144984	30534676	24.7
Bmp6 +/+ 2	53590124	41559304	29.01
Bmp6 +/+ 3	51516122	42258366	26.32
Bmp6 -/- 4	47897146	22799870	25.05
Bmp6 -/- 5	48728442	34295004	22.63
Bmp6 -/- 6	69383016	52728234	22.22
