# Supplementary material for: An intronic enhancer of Bmp6 underlies evolved tooth gain in sticklebacks
Source: PLoS Genet. 2018 Jun 14;14(6):e1007449. doi: 10.1371/journal.pgen.1007449 (PMC6019817; doi:10.1371/journal.pgen.1007449)
Supplement: S10 Table — GO term enrichment for a list of genes ranked by expression correlation with the first principal component of the Bmp6 wild-type and mutant tooth plate expression matrix. Shown are significant GO terms with an FDR q-value less than 1E-04. (PDF) [file pgen.1007449.s014.pdf]

GO Term	Description	FDR q-value
GO:0044421	extracellular region part	7.73E-12
GO:0005576	extracellular region	1.44E-11
GO:0005581	collagen trimer	8.85E-11
GO:0005615	extracellular space	1.03E-09
GO:0031012	extracellular matrix	2.58E-07
GO:0032502	developmental process	3.22E-07
GO:0044767	single-organism developmental process	6.04E-07
GO:0048646	anatomical structure formation involved in morphogenesis	6.57E-07
GO:0044707	single-multicellular organism process	8.78E-06
GO:0032501	multicellular organismal process	1.10E-05
GO:0098797	plasma membrane protein complex	1.90E-05
GO:0044459	plasma membrane part	2.25E-05
GO:0044724	single-organism carbohydrate catabolic process	2.48E-05
GO:0007155	cell adhesion	2.60E-05
GO:0016021	integral component of membrane	2.69E-05
GO:0044699	single-organism process	2.87E-05
GO:0022610	biological adhesion	2.97E-05
GO:0007166	cell surface receptor signaling pathway	3.75E-05
GO:0048856	anatomical structure development	4.04E-05
GO:0004872	receptor activity	4.57E-05
GO:0031224	intrinsic component of membrane	4.68E-05
GO:0016052	carbohydrate catabolic process	6.86E-05
GO:0001525	angiogenesis	7.27E-05
